# A Multimodal Pain Sentiment Analysis System Using Ensembled Deep Learning Approaches for IoT-Enabled Healthcare Framework

**DOI:** 10.3390/s25041223

**Published:** 2025-02-17

**Authors:** Anay Ghosh, Saiyed Umer, Bibhas Chandra Dhara, G. G. Md. Nawaz Ali

**Affiliations:** 1 Department of Computer Science & Engineering, University of Engineering & Management, Kolkata 700160, India; anay.ghosh1@gmail.com; 2 Department of Computer Science and Engineering, Aliah University, Kolkata 700156, India; saiyed.umer@aliah.ac.in; 3 Department of Information Technology, Jadavpur University, Kolkata 700032, India; bcdhara@gmail.com; 4 Department of Computer Science and Information Systems, Bradley University, Peoria, IL 61625, USA

**Keywords:** IoT, sentiment analysis, pain recognition, multimodal, CNN, audio, fusion

## Abstract

This study introduces a multimodal sentiment analysis system to assess and recognize human pain sentiments within an Internet of Things (IoT)-enabled healthcare framework. This system integrates facial expressions and speech-audio recordings to evaluate human pain intensity levels. This integration aims to enhance the recognition system’s performance and enable a more accurate assessment of pain intensity. Such a multimodal approach supports improved decision making in real-time patient care, addressing limitations inherent in unimodal systems for measuring pain sentiment. So, the primary contribution of this work lies in developing a multimodal pain sentiment analysis system that integrates the outcomes of image-based and audio-based pain sentiment analysis models. The system implementation contains five key phases. The first phase focuses on detecting the facial region from a video sequence, a crucial step for extracting facial patterns indicative of pain. In the second phase, the system extracts discriminant and divergent features from the facial region using deep learning techniques, utilizing some convolutional neural network (CNN) architectures, which are further refined through transfer learning and fine-tuning of parameters, alongside fusion techniques aimed at optimizing the model’s performance. The third phase performs the speech-audio recording preprocessing; the extraction of significant features is then performed through conventional methods followed by using the deep learning model to generate divergent features to recognize audio-based pain sentiments in the fourth phase. The final phase combines the outcomes from both image-based and audio-based pain sentiment analysis systems, improving the overall performance of the multimodal system. This fusion enables the system to accurately predict pain levels, including ‘high pain’, ‘mild pain’, and ‘no pain’. The performance of the proposed system is tested with the three image-based databases such as a 2D Face Set Database with Pain Expression, the UNBC-McMaster database (based on shoulder pain), and the BioVid database (based on heat pain), along with the VIVAE database for the audio-based dataset. Extensive experiments were performed using these datasets. Finally, the proposed system achieved accuracies of 76.23%, 84.27%, and 38.04% for two, three, and five pain classes, respectively, on the 2D Face Set Database with Pain Expression, UNBC, and BioVid datasets. The VIVAE audio-based system recorded a peak performance of 97.56% and 98.32% accuracy for varying training–testing protocols. These performances were compared with some state-of-the-art methods that show the superiority of the proposed system. By combining the outputs of both deep learning frameworks on image and audio datasets, the proposed multimodal pain sentiment analysis system achieves accuracies of 99.31% for the two-class, 99.54% for the three-class, and 87.41% for the five-class pain problems.

## 1. Introduction

The Internet of Things (IoT) [1] has transformed automation systems by integrating software, sensors, and electronic devices to collect and transmit data online without requiring human intervention. IoT-based devices are widely used in smart homes, traffic congestion management, and autonomous driving. In addition to these applications, IoT collaborates with artificial intelligence (AI) in the healthcare sector [2]. The healthcare industry is a key contributor to employment, revenue, and overall well-being, playing a crucial role in promoting a healthy society within smart cities. In healthcare, the IoT plays a pivotal role in automating systems such as patient monitoring and nurse call mechanisms [3]. For example, patient monitoring systems analyze data from smart devices, including thermometers, ECGs, pulse oximeters, and sphygmomanometers, to predict health conditions and notify medical staff to initiate timely interventions. The healthcare framework includes a wide range of components, such as medical professionals, hospitals, doctors, nurses, outpatient clinics, clinical tests, telemedicine, medical devices, machinery, and health insurance services [4]. Advances in technology have significantly enhanced healthcare through the adoption of electronic and mobile-based health services. Portable healthcare systems now offer patients daily health support, facilitating continuous monitoring and assistance [4]. These systems leverage speech recognition, gesture analysis, vocal tone, and facial expression analysis to remotely evaluate patients’ medical conditions. Gesture-based interaction and identification have become vital aspects of human–computer interaction (HCI), making e-healthcare systems more user-friendly and accessible for medical professionals.

Building on these advancements, this study introduces a multimodal pain sentiment analysis system (MPSAS) for remote patient monitoring, utilizing IoT-enabled medical sensors. The system integrates data from various sources, including audio and video, to categorize sentiments as positive, negative, or neutral. It also analyzes emotions such as happiness, anger, and frustration. By identifying these emotions, the system offers organizations valuable insights into sentiment, enabling them to make informed decisions to improve their products and services. This proposed sentiment analysis system enhances e-healthcare solutions, providing more cost-effective and efficient healthcare delivery. Facial expressions are known to effectively communicate unpleasant emotions, making them crucial for pain assessment [5]. In real-world applications, sentiment analysis systems remotely detect and interpret patients’ facial expressions to determine their emotional states [4]. For non-verbal patients, the American Society outlines a method for estimating pain through facial expressions. This method highlights a hierarchical framework for pain evaluation, where facial representation-dependent pain estimation is considered a valid approach. Payen et al. [6] found that facial expressions can play a crucial role in assessing pain in severely ill, unresponsive patients. In elderly individuals with serious mental health conditions, who are often unable to communicate verbally due to their deteriorating condition, facial expressions serve as a valuable tool for pain assessment. This approach is especially relevant for patients with advanced mental illnesses who cannot articulate their pain. McGuire and colleagues [7] concluded that individuals in non-verbal populations may not recognize or receive adequate treatment for their pain, primarily due to their inability to effectively communicate discomfort.

However, certain vulnerable groups—such as the physiologically immobile, terminally ill [7], critically ill but coherent, individuals in psychological distress, or those suffering from cancers affecting the head, neck, or brain—require dependable technological support to consistently monitor pain and alleviate the burden on healthcare professionals. Behavioral responses such as grimacing, eye closure, and wincing have been shown to strongly correlate with procedural pain, as highlighted in patient studies [8]. Pain assessment in clinical settings is vital for providing appropriate care and evaluating its effectiveness [9]. Traditionally, the gold standard for pain reporting involves patient self-reports through verbal numerical scales and visual analogue scales [10]. Additionally, sentiment analysis systems have been developed for conditions like Parkinson’s disease, depression, and for detecting intent and emotions from facial expressions, as well as for contextual semantic search. These systems utilize neural network techniques to analyze text, image, and video data to classify emotions and detect patterns. The principal contributions of this work are as follows:

- The proposed work develops a pain sentiment analysis system based on non-verbal communication (image or video) by utilizing multiple top-down deep learning models built on convolutional neural network (CNN) architectures. These models extract discriminative features from facial regions, enhancing feature representation for detecting varying levels of pain intensity.
- The proposed work also focuses on verbal communication (audio or speech) for pain sentiment analysis by employing handcrafted audio features as input, followed by deep learning models using CNN architectures to extract meaningful features for identifying different pain intensity levels.
- Performance improvements are achieved through extensive experimentation, including comparisons between batch processing and epoch cycles, data augmentation, progressive image resizing, hyperparameter tuning, and the application of pre-trained models via transfer learning.
- Post-classification fusion techniques are applied to enhance system accuracy by integrating classification scores from multiple deep learning models. This approach addresses challenges arising from variations in age, pose, lighting conditions, and noisy artefacts.
- Finally, a multimodal pain sentiment analysis system is developed by combining the results of image-based and audio-based models. This integration improves the system’s performance, enabling more accurate recognition of pain intensity and supporting real-time decision making in patient care.

Table 1 shows the list of abbreviations employed in this paper.

The structure of this research paper is as follows: Section 2 presents a review of the relevant literature. Section 3 provides a detailed explanation of the proposed methodology. Section 4 focuses on the evaluation of the conducted experiments and an overall analysis of the system. Finally, Section 5 summarizes the conclusions drawn from the study.

## 2. Related Work

This section reviews related work on pain sentiment analysis systems (PSASs) utilizing audio-based, image or video-based, and multimodal or hybrid prediction models. Acoustic or auditory pain refers to the discomfort experienced when exposed to sound. This pain can manifest in several ways: (i) hyperacusis, an increased sensitivity to everyday environmental sounds that can cause pain or discomfort even at low decibel levels; (ii) phonophobia, a severe fear or avoidance of loud noises, often accompanied by physical discomfort and anxiety; and (iii) tinnitus, a condition characterized by the perception of ringing, buzzing, or other phantom noises in the ears, which may not always be painful [11]. Furthermore, it has been demonstrated that acoustic elements of music, such as rhythm, tempo, and harmony, play a pivotal role in connecting pain relief with music therapy. A pioneering method for recognizing acoustic pain indicators in neonatal populations using audio features is proposed by Giordano et al. [12]. To investigate the relationship between self-reported pain intensity and measurable bioacoustic indicators in human vocalizations, Oshrat et al. [13] have developed a prosody-based approach. The work in [14] has introduced an automated process for evaluating pain based on paralinguistic speech features, aiming to enhance the objectivity and accuracy of pain diagnosis.

In computer vision, features extracted by various convolutional neural network (CNN) frameworks are employed for tasks such as emotion recognition and object identification. CNN-derived features have been observed to outperform handcrafted features in terms of performance. Recent advancements in deep learning have contributed significantly to solving complex challenges, establishing it as a leading approach in many domains. Automatic techniques for pain identification often demonstrate limited proficiency when relying on facial representations. In the domain of pain recognition systems using facial expressions, several widely adopted feature extraction methods are employed, such as active shape models and active appearance models [15], local binary pattern [16], and Gabor wavelets [17]. Existing approaches predominantly focus on facial representation-based pain detection and encompass deep learning and non-deep learning-based methodologies [18]. Significant advancements have been made in this research area in recent years. It has been observed that the classification outcomes are influenced by the extracted facial features [19]. Feature extraction is commonly performed using established methods. At the same time, classification is carried out using supervised machine learning techniques such as K-Nearest Neighbors, Support Vector Machines (SVMs), and Logistic Regression [20]. Nagarajan et al. [21] proposed a hybrid method combining Support Vector Machines (SVMs) and recurrent neural networks (RNNs) for detecting chronic pain. A technique to identify central neuropathic pain from EEG data, employing linear discriminant analysis and Linear SVMs, was developed in [22].

The classification of pain using a CNN framework was demonstrated in [23], and a comparative study utilizing bio-inspired algorithms for pain recognition was shown in [24]. A pain detection techniques was developed using the ‘Triplet Loss for Pain Detection’ technique to classify pain levels based on facial expressions in [25]. A two-stage method involving a 2D-CNN for frame-level feature extraction followed by an LSTM for sequence-level pain recognition was developed in [26]; similarly, a two-stage deep learning architecture incorporating the facial landmark localization and action-based analysis for pain detection was developed in [27]. Pain detection algorithms such as convolutional neural networks (CNNs), XGBoost, Extra Trees, and Random Forests are applied in [28]. In contrast, the Children Pain Assessment Neural Network was introduced in [29]. Rasha et al. [30] surveyed pain detection techniques, including multi-domain neural networks, CNN-LSTMs, and additive attributes. They also proposed a method for comparing feature extraction techniques, such as skin conductance level and skin conductance response, which retrieve features like the root mean square, local maxima and minima mean values, and mean absolute value for pain detection [31]. Kornprom et al. [32] developed a pain assessment method, introducing a pain intensity metric that incorporates tools such as the Sensory Scale, Affective Motivational Scale, Visual Analog Scale, and Observer Rating Scale. Leila et al. [33] created an automatic pain detection model using the VGG-Face model on the UNBC dataset. Jiang et al. [34] conducted experiments using the GLA-CNN approach for pain detection. Ehsan et al. [35] proposed a pain classification system utilizing video data from the X-ITE dataset and incorporated high-performance CNN frameworks such as GoogleNet and AlexNet.

Thiam et al. [36] presented a multimodal system for recognizing pain intensity by integrating audio and visual cues. Hossain [37] developed a multimodal system for assessing patient affective states, combining speech and facial expression modalities. Zeng et al. [38] surveyed, from a psychological perspective, human emotion perception through audio, visual, and spontaneous expressions. The work in [39] introduced a pain detection method that integrated unimodal and multimodal concepts. Deep learning algorithms can uncover intricate patterns within complex datasets, significantly enhancing feature extraction and classification processes [40]. Dashtipur et al. [41] proposed a multimodal framework based on context awareness for Persian sentiment analysis. Sagum et al. [42] introduced a sentiment measurement technique focused on emotion analysis using movie review comments. Rustam et al. [43] conducted a comparative study of various supervised machine learning methods for sentiment analysis of COVID-19-related tweets. The introduction of a neural network model combining CNN and BiLSTM architectures for emotion analysis was proposed in [44]. A pain detection method employing spatiotemporal data, using long short-term memory (LSTM) and spatiotemporal graph convolution networks (STGCNs), was developed in [45].

## 3. Proposed Methodology

**Figure 1:**
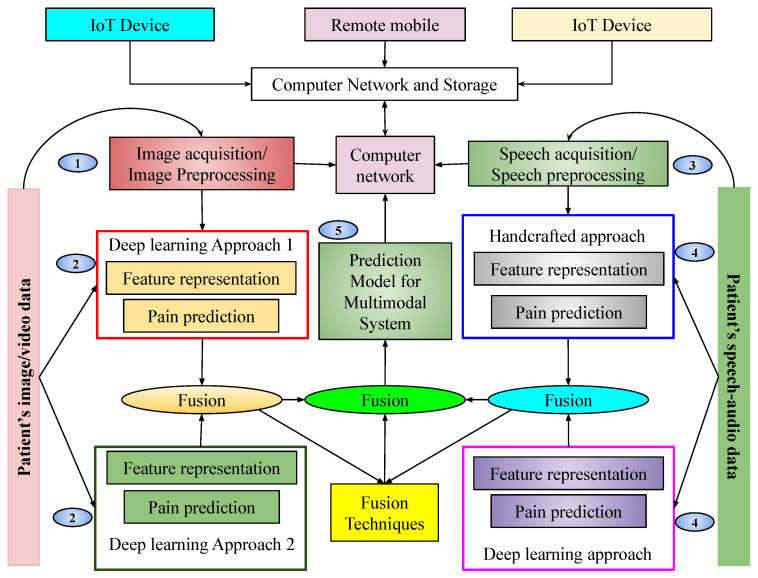
Pictorial representation of the proposed multimodal pain sentiment analysis system (PSAS) for smart healthcare framework.

This section outlines the step-by-step implementation of the proposed pain sentiment analysis system, including data preprocessing, feature extraction, model training, and evaluation. Facial expressions constitute a crucial factor in pain sentiment recognition, with the features of these expressions contributing to the identification of pain intensity. In cases where facial features are unavailable, audio-based sentiment features are utilized. Additionally, as facial expressions may vary based on individual pain perception, audio-based information can assist in determining the pain level. Therefore, combining both modalities enhances the overall effectiveness and competency of the proposed pain detection system. While several established pain detection algorithms exist, significant potential exists for further advancements in pain recognition techniques. In the current landscape, cloud-dependent mobile applications that rely on real-time data and live video processing are becoming increasingly popular. These applications typically consist of two components: the mobile front-end and cloud-based back-end. The cloud offers advancement in the processing of data and capabilities in computation. Our proposed method facilitates the execution of complex applications on devices with limited resources, optimizing performance and efficiency. This section describes a sentiment assessment method for the evaluation of pain levels based on facial expressions. The research integrates cache and computing facilities to provide essential factors for a smart portable healthcare framework. The system that has been proposed to recognize pain is structured into five key components, as illustrated in Figure 1.

This pictorial representation of our proposed system shows that there is a cloud network capable of storing some data. Various devices from outside the system communicate through that network. Our database is also connected to the cloud network. Our database mainly consists of images, videos, and audio. Before classification, images are fetched from the database, preprocessed, and reshaped based on the algorithm. After preprocessing, images are split into training and testing sets and fed into two CNN architectures. These CNN architectures are trained independently by using images considered for training purposes. Upon completion of training, the prediction models are obtained. These models generate scores for input samples, which are subsequently fused at the score level to arrive at a final prediction. The resulting prediction model is integrated into a network to enable remote pain intensity prediction. The detailed implementation is described in the following subsections.

### 3.1. Image-Based PSAS

**Figure 2:**
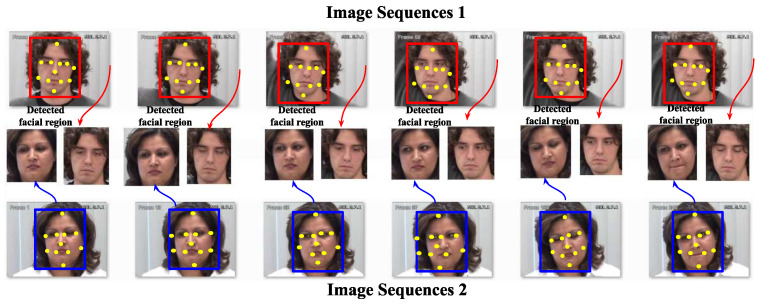
Detecting facial regions in input images for the image-based PSAS.

During the image acquisition phase, noise is often introduced, which reduces the textural information and detail of facial patterns essential for pain sentiment recognition. This noise can negatively impact the accuracy of the experiment. In the image preprocessing phase, a color image I is processed to extract the facial region for identifying pain emotion. A tree-structured part model (TSPM) [46] is employed, which is effective for handling various face poses. In this approach, the image is first divided into smaller parts, such as the nose, ears, eyes, and mouth. These parts are then connected using a tree-based data structure, and traditional computer vision techniques like Histogram of Oriented Gradients (HOG) and Scale-Invariant Feature Transform (SIFT) are applied to extract features from each part. The tree structure also defines the geometric relationships between the parts, which aids in handling pose variations of the face. Each part is assigned a score based on a scoring function that evaluates the spatial relationships between the parts. Optimization algorithms are employed to maximize the scoring function by training on annotated facial part locations and predicted scores, identifying several landmark points for each facial region. For frontal faces, the method detects 68 key facial landmarks, while for profile faces, 39 key landmarks are identified, focusing on features that are visible in both orientations. After detecting the facial landmarks, the extracted facial region undergoes normalization using bilinear image interpolation, resizing the face to dimensions of N×N. After facial region extraction, the feature computation tasks are performed. Here, several deep learning approaches are utilized to extract valuable and discriminating features. Distinct deep learning architectures are applied to the same dataset, and the resulting outputs are fused to move towards a final decision. The mechanism of detecting the facial region for the proposed pain identification technique is illustrated in Figure 2.

### 3.2. Image-Based Feature Representation Using Proposed Deep Learning Architectures

Visual feature extraction offers a reliable and consistent method for distinguishing between various objects. In applications such as emotion classification and facial expression identification, effective feature representation plays a pivotal role [47]. Deep learning-based convolutional neural network (CNN) architectures are utilized here [48] to learn features and classify facial region images into two or three pain-level classes. However, the performance of emotion recognition systems based on facial expressions is often hindered by overfitting, which results in sensitivity to factors such as pose, age, identity, and lighting variations. This approach addresses these challenges while reducing computational complexity. The direct classification of pain levels based on raw, unprocessed images is not feasible; hence, robust and efficient local-to-global feature representation schemes are applied for both feature synthesis and representation. CNN architectures have been highly effective in extracting diverse features from images and videos through advanced deep learning techniques [49]. In CNNs, various kernels process images using mixed convolution operations [50]. After the convolution operation, pooling techniques with weight sharing are employed to optimize the network training parameters. While structural and statistical dependent methodologies exist in the field of computer vision, state-of-the-art CNN-based feature representation techniques have proven to be more effective for facial expression-based pain sentiment analysis. These techniques particularly excel in handling challenges like articulation and occlusion in facial images captured under unconstrained conditions [49]. They enhance system performance by overcoming the difficulties posed by occlusion and variability in face images taken in uncontrolled environments.

The proposed system incorporates a sentiment analysis component that analyzes facial expressions using deep learning features while accounting for variables that influence the effectiveness of these features. In CNNs, the convolution operation serves as the foundation of the proposed network, forming the backbone of the CNN architecture and enabling effective image feature extraction. In this process, a filter or kernel of size t×t slides across the input image *I* to generate feature maps. At each position, the operation involves non-linear transformations and element-wise matrix multiplication, with the results producing feature map elements centered on the mask pattern. To optimize CNN architectures, parameter fine-tuning is employed, and several specialized layers are integrated to enhance performance:

1. **Convolutional layer:** This layer applies convolution to extract features from the input image. Non-linearity is introduced using an activation function, and the generated feature maps depend on learnable weights and biases.
2. **Max-pooling layer:** Max pooling reduces the size of feature maps by selecting the maximum value within each patch, thereby lowering computational complexity, maintaining translation invariance, and improving feature discrimination.
3. **Batch normalization layer:** This layer normalizes intermediate outputs, stabilizing and speeding up training. It also allows the use of higher learning rates effectively.
4. **Dropout layer:** Acting as a regularization technique, dropout prevents overfitting by randomly deactivating neurons during training, encouraging the network to generalize better.
5. **Flatten layer:** This layer converts multi-dimensional feature maps into a one-dimensional vector, bridging the convolutional layers and the fully connected layers.
6. **Fully connected layers:** These layers aggregate the extracted features into a final vector, with the size corresponding to the number of class labels. The output is used for classification.
7. **Output layer:** The final layer employs the Softmax function to perform classification and determine the output probabilities for each class.

The proposed feature representation schemes outline two key functions of the CNN baseline model: Extraction of features and performing classification. The CNN models are structured with seven to eight deep image perturbation layers, each designed to enhance performance. The ReLU activation function is applied as the introduction of non-linearity is required; for the reduction of spatial dimensionality max pooling is applied and captures key features; and for accelerating the training procedure and upgrading of generalization, batch normalization is employed. After being flattened into a one-dimensional vector, the resulting feature maps are processed through two fully sequentially connected layers and finally, for multi-class classification, a Softmax layer is used. To improve the performance of the CNN model, progressive image resizing techniques are applied, adding new levels to the model. These techniques help reduce overfitting, tackle imbalanced data, and optimize computational resource usage.

**Figure 3:**
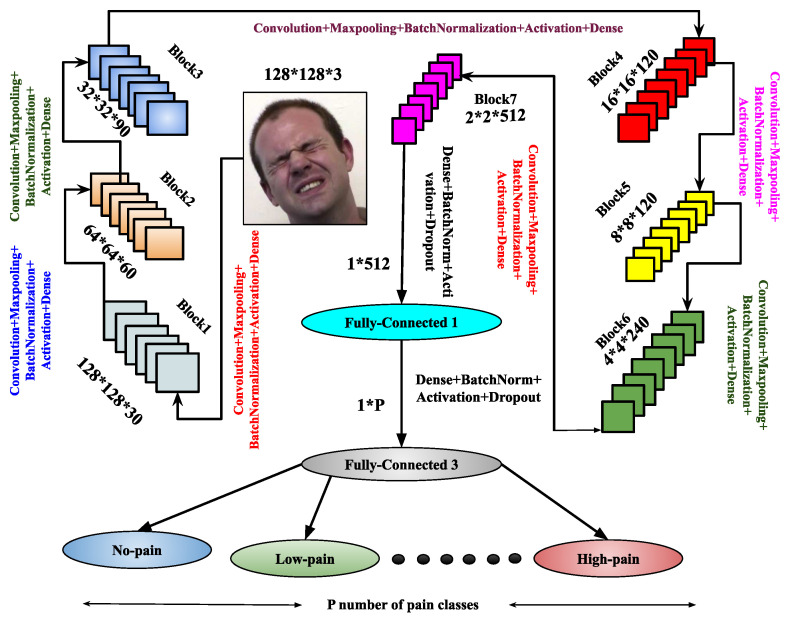
Demonstration of the $CNN_{A}$ architecture for image-based PSAS.

**Figure 4:**
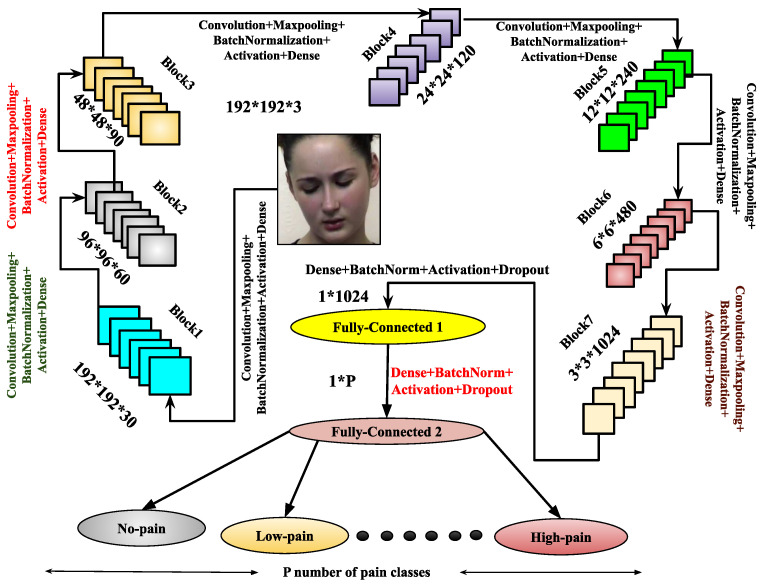
Illustration of the $CNN_{B}$ architecture.

The proposed hybrid design of the CNN architectures enables the extraction of shape along with texture information from images. The recommended system utilizes textual and shape-based features to provide both geometrical and appearance-based descriptions. The primary drawback of the CNN model, which is thought to be significant computation power requirements, can be mitigated by using graphical processing units. Graphical processing units are primarily designed to handle high-volume computations, encompassing a wide range of representation and concept levels, including transfer learning, pre-training, data patterns, and techniques for fine-tuning convolutional neural networks. In this work, during training the proposed CNN frameworks, some large databases are used to train the architectures by weight adjustment of the network depending on the number of defined pain classes. So, this research proposes two CNN frameworks: $CNN_{A}$ and $CNN_{B}$. The respective trained models generated from these CNN architectures are $f_{CNN-A}$ and $f_{CNN-B}$. For a better understanding and transparency regarding the models, the specifics of these architectures with their use of input–output hidden layers, convoluted image output shape, and parameters produced at each layer are further described. These are shown in Figure 3 and Figure 4, respectively. Similarly, Table 2 and Table 3 illustrate the list of parameters required for training these CNN architectures.

Once training is completed, the trained model $f_{CNN-A}$ is derived from the $CNN_{A}$ architecture. To enhance feature representation, another architecture, $CNN_{B}$, is introduced. It comprises eight blocks, each containing a convolutional layer, max-pooling, batch normalization, and dropout layers. After these blocks, the architecture includes three flattened layers, each followed by a dense layer with batch normalization, the ReLU activation function, and a dropout layer. The final layer is a dense layer used for output classification. The $CNN_{B}$ model is compiled using the Adam optimizer, and the trained model $f_{CNN-B}$ is derived upon completion of training. Both the $CNN_{A}$ and $CNN_{B}$ architectures are designed to enhance performance through improved feature abstraction. These architectures significantly contribute to the image-based pain sentiment analysis system by providing refined and efficient models.

(i) $Scheme_{1}$: The first factor involves incremental image resizing. The input layers in $Scheme_{1}$ continuously receive images as input data. Convolution operations are performed using multiple masks or filter banks, generating various convolved images or feature maps based on the filters [51]. As image size increases, convolution operations become more computationally demanding due to the growing number of filters. The parameters within these filter sets are considered as the refined weights. For max-pooling layers, the number of parameters in the network also impacts computational overhead. It has been observed that analyzing patterns from images at multiple resolutions enhances the feature representation capabilities of CNN frameworks using deep neural network techniques. Adding layers to the architecture as image resolution increases allows for a deeper analysis of hidden patterns in the feature maps. Motivated by this, multi-resolution facial image techniques have been implemented using various CNN architectures and layers. Specifically, two different image resolutions of the normalized facial region *F* are considered in $Scheme_{1}$, where $F_{k\times k}$ is down-sampled to $F_{k_{1}\times k_{1}}$ and $F_{k_{2}\times k_{2}}$, with $k>k_{2}>k_{1}$.

(ii) $Scheme_{2}$: The second aspect involves utilizing transfer learning combined with fine-tuning techniques. Transfer learning facilitates the development of discriminant features essential for the pain sentiment analysis system (PSAS), demanding higher-order feature representation. In this process, certain layers and their parameters are frozen, while others are retrained to reduce computational complexity. This approach enables a model trained for one problem to be effectively applied to related tasks. In the proposed model, transfer learning integrates CNN-based deep learning, utilizing layers and weights from a pre-trained model. This reduces training time and minimizes generalization errors.

(iii) $Scheme_{3}$: The third factor involves the application of fusion techniques. Trained models generate features or scores, which are subsequently transformed into new sets of features or scores. Fusion techniques are employed to combine classification scores from trained deep learning models, to enhance the overall performance of the proposed pain identification system. In this particular research activity, the scores are fused after the completion of the classification with the help of *product-of-score*, *weighted-sum-of-score*, and *sum-of-score*. For instance, consider two trained deep learning models, $\zeta_{1}$ and $\zeta_{2}$, which solve an *L*-class problem and generate classification scores $l_{1}\in R^{1\times L}$ and $l_{2}\in R^{1\times L}$ for a test sample *a*. Fusion methods are applied as follows:

- $l^{p}=\left(l_{1}\times l_{2}\right)\in R^{1\times L}$ (*product of score*);
- $l^{w}=\left(\rho_{1}\times l_{1}+\rho_{2}\times l_{2}\right)\in R^{1\times L}$ (*weighted sum of score*);
- $l_{s}=\left(l_{1}+l_{2}\right)\in R^{1\times L}$ (*sum of score*).

Here, $\rho_{1}$ and $\rho_{2}$ are weights assigned to models $\zeta_{1}$ and $\zeta_{2}$, respectively, with $\rho =\rho_{1}+\rho_{2}$. Experimentally, it has been observed that if $\zeta_{1}$ outperforms $\zeta_{2}$, then $\rho_{1}>\rho_{2}$, and vice versa.

### 3.3. Audio-Based PSAS

**Figure 5:**
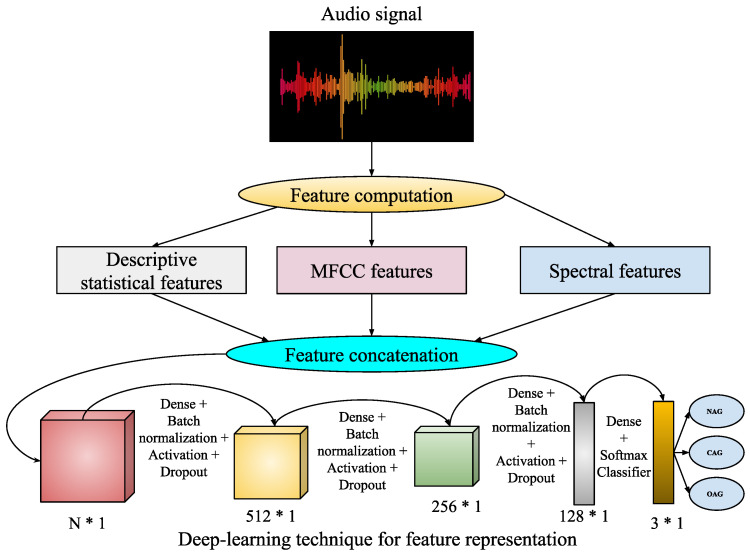
Executed $CNN_{1}$ framework.

In the digital world, audio-based media content is kept in the form of signal processing, where dealing with, processing, and manipulation of audio signals are performed for various applications such as speaker recognition, gender classification, age classification, disease detection, entertainment, and health care. Converting analogue signals to digital signals in audio files is challenging for various types of media content, such as text, images, videos, and audio. The chances of unwanted noise introduction and unbalanced time–frequency distribution make audio-based recognition systems much more challenging. In this work, an audio-based speaker’s sentiment analysis is performed to detect the intensity levels of their body-part pain sentiments. The purpose of an audio noise reduction system is to remove noise from audio signals, such as speech. There are two types of these systems: Complementary and non-complementary. Before properly recording the audio stream, complementary noise reduction entails compressing it. Conversely, single-ended, or non-complementary, noise reduction works well for lowering noise levels [52]. Digital and analogue devices are vulnerable to noise because of specific characteristics. It is possible to extract different aspects more precisely when noise is removed from audio signals. In this work, we have utilized filtered audio signals to extract audio features for pain sentiment analysis. Here, identifying pain sentiment levels is based on analyzing the types of emotions a person has during human–computer-based interaction in the healthcare system. Fortunately, facial expressions are analyzed or recognized in image- or video-based emotion recognition. Still, in real-time scenarios, video-based media might record the vocal emotions of a patient in the online healthcare framework. Then, there will be the requirement for audio-based emotion recognition systems. Here, feature representation using the deep learning-based approach for audio-based classification is demonstrated in Figure 5.

This work utilizes speaker audio recordings for audio-based pain sentiment evaluation. The audio data are provided in waveform format, consisting of a sequence of bytes representing audio signals over time. Hence, for analyzing these sequences of bytes in terms of numeric feature representation, some feature extraction techniques are employed for feature computation from the waveform audio files. These feature extraction techniques are described as follows:

- Statistical audio features: Here, the audio signal initially undergoes a frequency-domain signal analysis using fast Fourier transformation (FFT) [53]. The computed frequencies are subsequently employed to calculate descriptive statistics, including mean, median, standard deviation, quartiles, and kurtosis. The magnitude of frequency components is used to calculate energy and root mean square (RMS) energy. Here, the use of FFT transforms the audio signals into frequency components, which are used to analyze audio characteristics, such as to measure tone, pitch, and spectra content. So, the measure of descriptive statistics gives a general representation of these audio characteristics, whereas energy and RMS energy distinguish low and high loudness intensities with variations in their magnitudes.
- Mel-frequency cepstral coefficients (MFCCs): These represent a well-known audio-based feature computation technique [54], where the derived frequency components from FFT undergo a log-amplitude transformation. Then, the mel scale is introduced to the logarithm of the amplitude spectrum, discrete cosine transformations (DCTs) are applied on the mel scale, and finally 2–13 DCT coefficients are kept. The rest are discarded during feature computations. Here, the extracted final features of MFCCs represent the compactness (i.e., better for discriminating audio signals between subjects), and apply to domain relevance for diverse applications of audio-based recognition systems.
- Spectral features: Various spectral features like spectral contrast, spectral centroid, and spectral bandwidth are analyzed from the audio files. These spectral features [55] are related to the spectrograms of the audio files. The spectrogram represents the frequency intensities over time. It is measured from the squared magnitude of the STFT [56], which is obtained by computing an FFT over successive signal frames. These types of features can extract richness and harmonicity from the audio signals.

So, using these above feature extraction techniques, $d_{1}$-dimensional statistical features ($f_{1}$), $d_{2}$-dimensional MFCC features ($f_{2}$), and $d_{3}$-dimensional spectral features ($f_{3}$) are extracted from each speech audio file. Then, these extracted feature vectors are combined such that $f_{audio}=\left(f_{1},f_{2},f_{3}\right)$. Now, $f_{audio}\in R^{1\times d}$, $d=d_{1}+d_{2}+d_{3}$, undergoes the proposed deep learning-based feature representation followed by classification for speech audio. During feature representation, the *d*-dimensional feature vector is transformed to a 512-dimensional feature, then 256- to 128-dimensional features. Finally, during classification, these are mapped into a three-class audio-of-pain problem. The list of parameters required for this audio-based deep learning technique is demonstrated in Table 4.

## 4. Experiments

The experiments in this study are conducted in two phases. An image-based pain sentiment analysis system is developed in the first phase, while the second phase focuses on an audio-based pain sentiment analysis system. For the image-based or video-based system, facial expressions are analyzed by extracting the facial region and the area of interest during preprocessing. The extracted images are resized to 128×128×3 or 192×192×3 to create a normalized facial region F. The $CNN_{A}$ architecture processes images of size 128×128×3, while $CNN_{B}$ handles images of size 192×192×3. Both architectures employ a 50–50% training–testing split, where 50% of the images or videos from each pain class are used for training and the remaining 50% for testing. The hyperparameter settings for these CNN architectures are detailed in Table 2 and Table 3, with both architectures utilizing the ‘Softmax’ classifier and the ‘Adam’ optimizer. Similarly, the audio-based pain sentiment analysis system (PSAS) employs a deep learning-based feature representation approach, as described in the above section, utilizing a one-dimensional CNN architecture. Like the image-based system, the PSAS follows a 50–50% training test protocol to evaluate its performance. The proposed pain sentiment analysis system was implemented using Python 3.11.5 version for this research. The implementation was carried out on a system featuring an Intel Core i7 7th-generation processor (7700 K) (3.20 GHz), 64 GB DDR4 RAM, and 12 GB NVIDIA TITAN XP GPU running on the Windows 10 operating system. Key Python libraries, such as Keras [57] and Theano [58], were utilized to support the development of deep learning-based convolutional neural network (CNN) architectures and techniques, which form the foundation of the system.

### 4.1. Used Databases

The evaluation of the proposed system was conducted using one audio-based, two image-based, and one video-based dataset. The audio dataset, “Variably Intense Vocalizations of Affect and Emotion Corpus (VIVAE)” [59], and the image datasets, the UNBC-McMaster Shoulder Pain Expression Archive [60] and the 2D Face Set Database with Pain Expressions (D2) [61], were utilized for testing.

**Figure 6:**
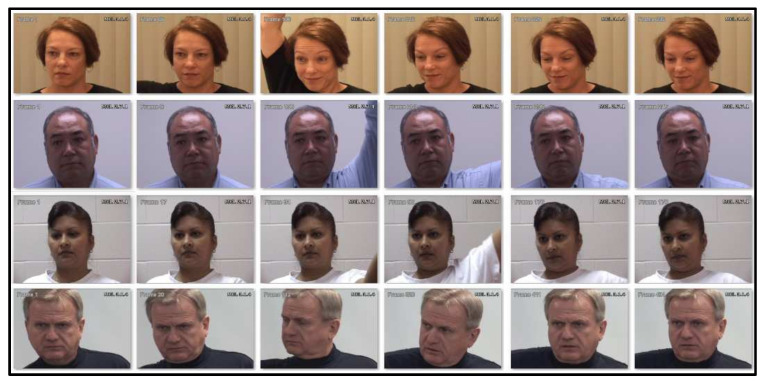
Examples of some image samples from UNBC-McMaster [60] database.

The first dataset is the UNBC-McMaster Shoulder Pain Expression Archive dataset, which comprises recordings of 129 participants (66 females and 63 males), all diagnosed with shoulder pain by physiotherapists at a clinic. The videos were captured at McMaster University, documenting participants with various shoulder pain conditions such as rotator cuff injuries, bone spur, arthritis, subluxation, dislocation, tendinitis, bursitis, impingement syndromes, and capsulitis. Images were extracted from these videos and categorized by pain intensity into classes ranging from ’no pain’ to ’high-intensity pain.’ For this study, the dataset was organized into two-class and five-class problems of classification. For the three class problems of classification, the involved labels are no pain ($R_{1}$), which indicates the non-aggressive (NAG) class of data; low pain ($R_{2}$), for implying the covertly aggressive (CAG) class of data; and the last one is high pain ($R_{3}$), for indicating the overtly aggressive (OAG) class of data. Representative samples from this dataset are displayed in Figure 6, and a detailed description of the dataset’s two-class and three-class classifications is provided in Table 5.

**Figure 7:**
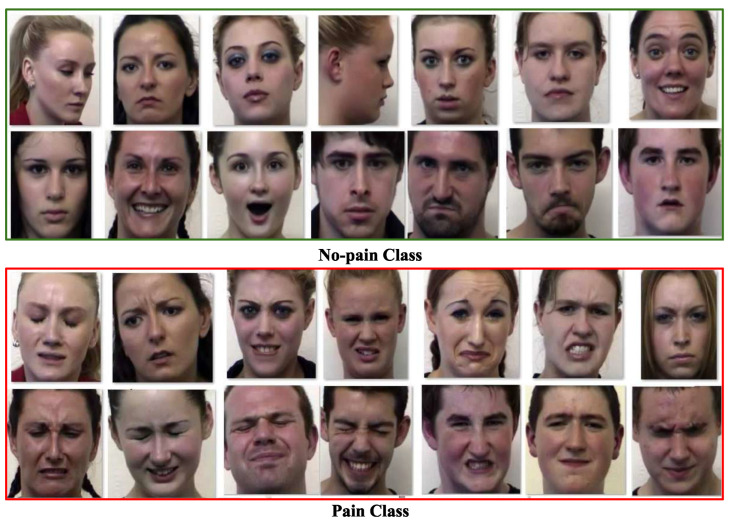
Examples of some image samples from 2DFPE [61] database.

The second image-based dataset, the 2D Face Set Database with Pain Expressions (2DFPE) [61], includes 599 images collected from 13 women and 10 men. This dataset primarily focuses on a two-class classification problem, where 298 images are labeled as ‘no pain’ ($P_{1}$) and the remaining 298 images as ‘pain’ ($P_{2}$). A comprehensive description of this database is available in Table 5, with sample images shown in Figure 7.

**Figure 8:**
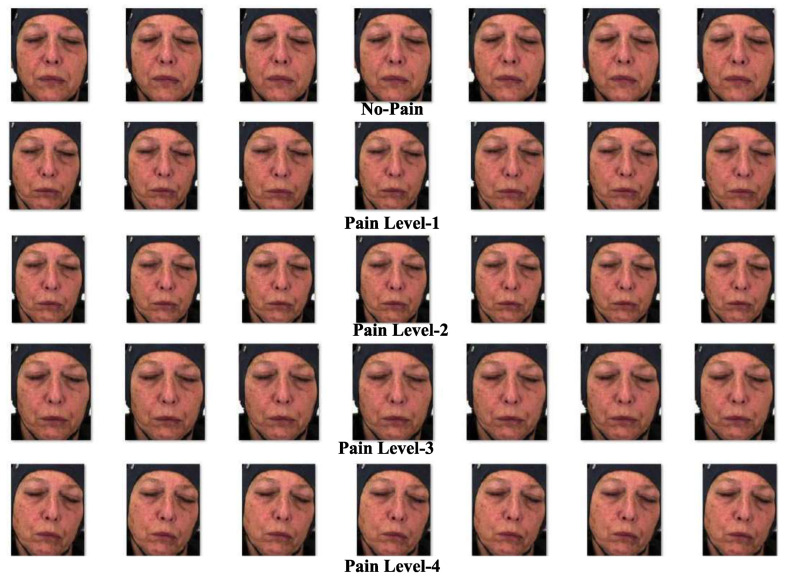
Samples of some image specimens from BioVid Heat Pain Database [62].

The BioVid Heat Pain Database [62] is the third dataset used in this research, and it is based on video samples. The dataset consists of twenty videos per class and five distinct classes for each of the 87 subjects. The five distinct data categories in the dataset are part A through part E, with part A selected for this work. From the list of 87 subjects, five subjects were chosen for part A, with 50 videos taken under consideration for each subject. The number of selected videos for every individual subject representing each label was ten; however, the total number of labels was five. As a whole, a total of 250 videos of a length of 5.5 s was considered; each of these videos has a total of 138 frames. The frames or images from each video were extracted and assigned one of five distinct labels, ranging from “no pain” to “extreme-intensity pain.” These labels correspond to the following pain intensity categories: baseline ($S_{1}$), intensity of ‘pain amount 1’ ($S_{2}$), intensity of ‘pain amount 2’ ($S_{3}$), intensity of ‘pain amount 3’ ($S_{4}$), and intensity of ‘pain amount 4’ (${\mathrm{PI}}_{5}$). Example images from the BioVid Heat Pain Database are shown in Figure 8. A description of the BioVid Heat Pain dataset, including its correlation to image specimens for particular pain labels, is provided in Table 5.

The fourth dataset utilized in this research is a speech audio database known as the Variably Intense Vocalizations of Affect and Emotion Corpus (VIVAE) [59]. The VIVAE database comprises non-speech emotion vocalizations recorded from human participants. It contains 1085 audio recordings collected from 11 speakers, each expressing various emotions, including fear, anger, surprise, pain, achievements, and sexual pleasure. For each emotion, recordings were captured across four intensity levels: ‘low’, ‘moderate’, ‘peak’, and ‘strong’. This study focuses on detecting pain intensity, categorizing the recordings into three main classes: Non-aggressive (NAG), where the intensity of pain is zero ($T_{1}$); covertly aggressive (CAG), representing a low pain intensity ($T_{2}$); and overtly aggressive (OAG), corresponding to a high pain intensity ($T_{3}$). For simplicity, recordings labeled with ‘low’ and ‘moderate’ intensities are grouped under the NAG class, ‘peak’ is assigned to the CAG class, and ‘strong’ is classified as OAG. The emotions in the dataset are thus distributed across these three pain intensity classes based on their intensity levels. Specifically, the database includes 530 audio files in the NAG class, 272 files in the CAG class, and 282 files in the OAG class. Experiments with the proposed system evaluate its performance using distinct training and testing sets derived from these recordings.

### 4.2. Results for Image-Based PSAS

The image-dependent pain sentiment analysis system (PSAS) consists of two main stages: (i) preprocessing of the image, and (ii) feature learning along with classification. In the preprocessing phase, the facial region, which is the area of interest, is first extracted. The images are then resized to 128×128×3 or 192×128×3 for the normalized facial region F. After these transformations, the images with facial expressions from the training sample of the dataset are input to the proposed CNN architectures, as shown in Figure 3 and Figure 4. Specifically, the $CNN_{A}$ architecture processes input images of size 128×128×3, while the $CNN_{B}$ architecture handles images of size 192×192×3. Within these CNN architectures, feature extraction and pain sentiment classification are performed.

The primary focus of the training phase is to optimize the performance of these architectures. Once trained, the models $f_{CNN-A}$ and $f_{CNN-B}$ are tested on the test samples to assess their performance. The experimental process begins by testing the $CNN_{A}$ architecture with a 50–50% training–testing split, applied to both the 2DFPE database (two classes of pain level) and the UNBC database (three classes of pain level).

**Figure 9:**
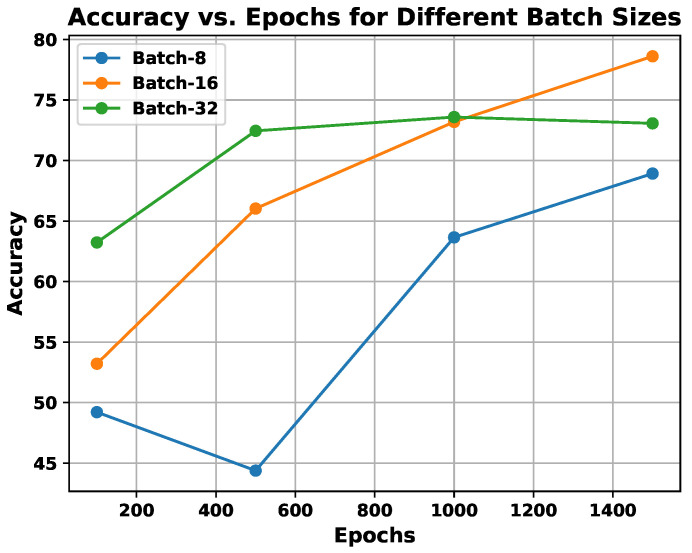
Demonstration of utilization of $Scheme_{1}$ experiments, exploring the effect of batch size vs. epochs on the proposed system’s performance.

- **$Scheme_{1}$ experiment:** This experiment is centered around parameter learning, focusing on batch size and epoch count, which are critical factors for the performance of any CNN architecture. Both the batch size and the number of epochs have a substantial impact on the ability of the network to learn when training on the provided samples. One of the primary tasks in deep CNN models is optimizing the learning of weight parameters. To improve this process, this study identifies an optimal trade-off between a batch size of 16 and 1500 epochs, aiming to enhance the outcomes of the proposed system. The results of an experiment comparing the effects of batch size and epoch count on system performance are presented in Figure 9 using the UNBC two-class dataset. As shown in the figure, performance improves with a batch size of 16, and the epoch count varies between 1000 and 1500.

**Figure 10:**
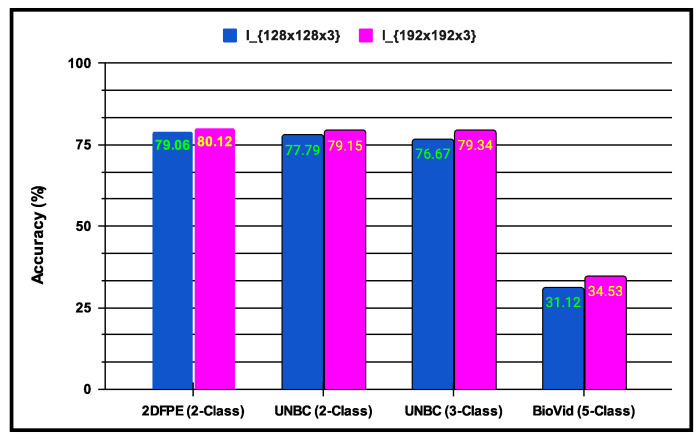
Demonstration of $Scheme_{1}$ experiments performing multi-resolution image analysis on the performance of the proposed system.

In $Scheme_{1}$, the experiments are conducted to incorporate multi-resolution facial images using progressive image resizing. This approach leverages multi-resolution image analysis to take advantage of progressive image scaling, allowing images of two different sizes to be processed by the $CNN_{A}$ and $CNN_{B}$ architectures. Compared to the standard CNN architecture performance, $Scheme_{2}$ offers several advantages: (i) networks can be trained with images having diverse dimensions, such as from low to high; (ii) representations of hierarchical features are obtained for each image, leading to enhanced texture pattern discrimination; and (iii) overfitting issues are minimized. The performance of multi-resolution image analysis in the proposed PSAS is illustrated in Figure 10 for the 2DFPE database (2 classes of pain level), UNBC database (2 classes of pain level), UNBC database (3 classes of pain level), and BioVid Heat Pain dataset (5 classes of pain level). The figure demonstrates that the $f_{CNN}^{Scheme_{1}}$ model effectively addresses overfitting and enhances the proposed system’s overall performance.

- **$Scheme_{2}$ experiment:** This experiment investigates the influence of applying transfer learning with fine-tuning on the performance of the two new CNN models trained on the same image data domain. Fine-tuning involves adjusting the weights of an existing CNN architecture, which reduces training time and improves results. Transfer learning is an improvised version of machine learning, in which any model is trained to fulfill one specific task and utilized as the base for constructing a new framework for a similar task. In this experiment, two transfer learning strategies are tested: (i) the first approach trains both CNN architectures, $CNN_{A}$ and $CNN_{B}$, from scratch using the appropriate image sizes; and (ii) the second approach retrains the models using pre-trained weights from both architectures. The results indicate that the retrained models outperform those trained from scratch. Table 6 displays the outcome of the CNN architectures, where the evaluation is performed based on accuracy and *F1-score*. The findings suggest that approach 1 improves the performance of the proposed PSAS.

**Table 6:** Proposed PSAS’s performance when applying approach 1 (A1) & approach 2 (A2) of fine-tuning and transfer learning.

Dataset	Using $I_{128\times 128\times 3}$			
	Acc. (%)	F1-Score	Acc. (%)	F1-Score
2DFPE (2 classes of pain level)	75.27	0.7563	72.22	0.7041
UNBC (2 classes of pain level)	83.11	0.8174	83.16	0.7918
UNBC (3 classes of pain level)	82.44	0.8437	82.36	0.8213
BioVid (5 classes of pain level)	34.11	0.3304	32.73	0.3016
Dataset	Using $I_{192\times 192\times 3}$			
2DFPE (2 classes of pain level)	75.81	0.7533	76.14	0.7524
UNBC (2 classes of pain level)	84.12	0.7916	83.53	0.7414
UNBC (3 classes of pain level)	82.46	0.8334	82.37	0.8103
BioVid (5 classes of pain level)	37.45	0.3571	36.89	0.3518

- **$Scheme_{3}$ experiment:** In this experiment, various fusion techniques are applied to the classification outcome scores produced by the trained $CNN_{A}$ and $CNN_{B}$ architectures. The classification results are obtained from deep learning features, and a post-classification fusion approach is employed. The classification scores generated by each CNN model are then processed using the *weighted-sum rule*, *product rule*, and *sum rule* fusion methodologies. Table 7 presents the fused outcome performance of the proposed system based on different combinations of these fusion methods. The results show that the system performs optimally when using the *product rule* fusion method, which integrates both CNN architectures, surpassing the performance of the *weighted-sum rule*, *sum rule*, and other techniques. The proposed system achieves accuracy rates of 85.15% for the two-class UNBC dataset, 83.79% for the three-class UNBC dataset, and 77.41% for the two-class 2DFPE dataset, which will be referenced in the subsequent section.

**Table 7:** Performance outcome of the proposed PSAS in terms of Acc. (%) when applying fusion methods to different CNN architectures; CPL means classes of pain levels.

Method	2DFPE (2-CPL)	UNBC (2-CPL)	UNBC (3-CPL)	BioVid (5-CPL)
$CNN_{A}$ (A1)	75.27	83.11	82.44	34.11
$CNN_{A}$ (A2)	72.22	83.16	82.36	32.73
Sum rule (A1 + A2)	75.56	83.47	82.51	34.67
Product rule (A1 × A2)	76.67	84.11	83.42	35.13
Weighted-sum rule (A1A2)	76.37	84.11	83.15	35.23
$CNN_{B}$ (A1)	75.81	84.27	82.46	37.45
$CNN_{B}$ (A2)	76.14	83.53	82.37	36.89
Sum rule (A1 + A2)	76.81	84.69	82.74	37.89
Product rule (A1 × A2)	**77.41**	**85.15**	**83.38**	**38.04**
Weighted-sum rule (A1A2)	77.23	85.07	83.17	37.83

**Figure 11:**
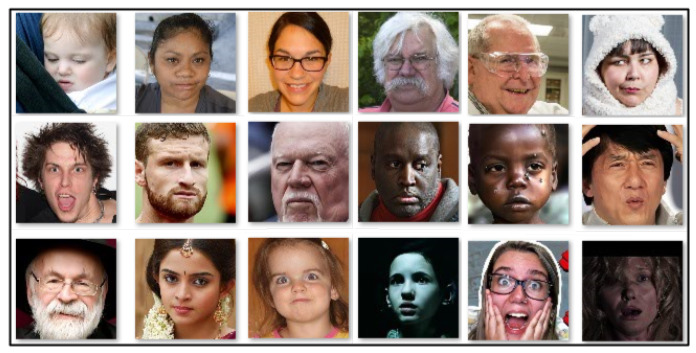
Demonstration of some image samples of AffectNet dataset [64] with ethnic diversity and variations in age among the subjects to validate the robustness of the proposed methodology.

Facial expressions from people of different ethnicities influence the performance of facial expression recognition systems. To address this, the proposed system incorporates feature representation methods specifically designed for non-verbal communication, which aim to enhance performance across diverse ethnic groups and across age variations. While facial expressions are largely universal—encompassing seven universally recognized basic expressions [63]—the challenge of ethnicity impacting recognition accuracy remains significant, primarily due to the scarcity of pain datasets explicitly designed to encompass diverse ethnicities across different age groups. Here, the proposed system’s robustness in identifying facial expressions is validated across ethnicities with variations in age; an experiment was conducted using the challenging AffectNet [64] facial expression dataset, which is composed of 29,042 image samples spanning eight facial expression categories: anger, contempt, disgust, fear, happy, neutral, sad, and surprise. Figure 11 illustrates examples from this dataset. The facial expression recognition system is built using the same deep learning architectures (Figure 3 and Figure 4) and training–testing protocols employed in the proposed pain sentiment analysis system (PSAS). The system’s performance is compared with competing methods used for PSA, and the results are summarized in Table 8. The findings show that the proposed feature representation methods achieve superior performance, even on ethnically diverse data and data with variations in age in the facial expression dataset. This demonstrates the system’s robustness and adaptability in accurately recognizing and analyzing facial expressions across varied demographic groups and age ranges, and also in the context of pain recognition systems.

- **Comparison with existing systems for the image-based PSAS:** The effectiveness of the proposed system has been benchmarked against several state-of-the-art techniques across multiple datasets: the two-class 2DFPE database; the UNBC database considering the two-class and three-class classification problems; and the BioVid database considering the five-class classification problem. Feature extraction techniques, including local binary pattern (LBP) and Histogram of Oriented Gradients (HoG), as utilized in [65,66], were employed. For each image, the feature representation was generated using a size of 192×192 pixels, and the images were divided into nine blocks for both the LBP and HoG methods. From each block, a 256-dimensional LBP feature vector [65] and an 81-dimensional HoG feature vector [66] were extracted, yielding 648 HoG features and 2304 LBP features per image. For deep feature extraction, methods such as ResNet50 [67], Inception-v3 [68], and VGG16 [69] were employed, each using an image size of 192×192. Transfer learning techniques were applied by training each network on samples from either the two-class or three-class problems. Features were then extracted from the trained networks, and neural network-based classifiers were used for the classification of test samples. Competing approaches from Lucey et al. [60], Anay et al. [70], and Werner et al. [71] were also implemented, using the same image size of 192×192. The results for these techniques were derived using the same training and testing protocol as the proposed system. These results are summarized in Table 9, which shows that the proposed system outperforms the competing techniques.

**Table 8:** Performance comparison of the image-based facial expression recognition system using the same deep learning techniques (Figure 3 and Figure 4) and training–testing protocols employed in the proposed pain sentiment analysis system (PSAS).

	8-Class AffectNet	
Method	Acc. (%)	F1-Score
Anay et al. [70]	56.45	0.5352
HoG	43.67	0.4018
Inception-v3	51.71	0.4731
LBP	40.47	0.3659
Lucey et al. [60]	55.04	0.5345
ResNet50	41.19	0.3984
VGG16	43.26	0.4221
Werner et al. [71]	56.87	0.5339
Proposed	**61.67**	**0.6034**

**Table 9:** Performance comparison of image-based sentiment analysis system using 2-class, 3-class, and 5-class pain sentiment problems.

	3-Class UNBC-McMaster		2-Class UNBC-McMaster		2-Class 2DFPE		5-Class BioVid	
Method	Acc. (%)	F1-Score	Acc. (%)	F1-Score	Acc. (%)	F1-Score	Acc. (%)	F1-Score
Anay et al. [70]	82.54	0.7962	83.71	0.8193	75.67	0.7391	27.33	26.89
HoG	63.19	0.6087	73.29	0.6967	68.61	0.6364	24.34	24.17
Inception-v3	72.19	0.6934	76.04	0.7421	63.89	0.6172	23.84	22.31
LBP	65.89	0.6217	75.92	0.7156	67.34	0.6386	26.10	25.81
Lucey et al. [60]	80.81	0.7646	80.73	0.7617	72.58	0.6439	29.46	28.63
ResNet50	74.40	0.7145	77.79	0.7508	63.78	0.6154	28.92	28.78
VGG16	74.71	0.7191	76.16	0.738	61.98	0.5892	25.64	23.55
Werner et al. [71]	75.98	0.7233	76.10	0.7531	66.17	0.6148	31.76	29.61
Proposed	**83.38**	**0.8174**	**85.07**	**0.8349**	**77.41**	**0.7528**	**37.42**	**35.84**

Table 9 provides a comparison of the performance outcome of the sentiment analysis system based on images beyond both the two-class and three-class pain sentiment analysis tasks. All experiments were conducted using identical training and testing protocols. The proposed two-class classification model (A) was quantitatively compared to several competing methods: Anay et al. [70] (B), HoG [66] (C), Inception-v3 [68] (D), LBP [65] (E), Lucey et al. [60] (F), ResNet50 [67] (G), VGG16 [69] (H), and Werner et al. [71] (I). A comparative statistical evaluation of the proposed system (A) against the competing approaches ({B, C, D, E, F, G, H, I}) is provided in Table 10. A one-tailed *t*-test [72] was performed using the t-statistic to analyze the results. This test involved two hypotheses: (i) H0 (null hypothesis): $\mu_{A}\leq \mu_{\left\{B,C,D,E,F,G,H,I\right\}}$ (indicating that the proposed system (A) demonstrates comparable results to the competing approaches on average); and (ii) H1 (alternative hypothesis): $\mu_{A}>\mu_{\left\{B,C,D,E,F,G,H,I\right\}}$ (suggesting that the proposed system (A) performs better than the competing methods on average). Rejection of the null hypothesis occurs if the *p*-value is less than 0.05, signifying that the proposed system (A) demonstrates better performance than the competing methods. For the quantitative comparison, each test dataset from the employed databases was divided into two sets. For the $D_{2}$ database, there were 298 test images (set 1: 149; set 2: 149). For the UNBC (two-class/three-class) problem, there were 24,199 test samples (set 1: 12,100; set 2: 12,099). The performance of the proposed and competing methods was evaluated using these test sets. The results, shown in Table 10 confirm that the alternative hypothesis is supported in all cases, and the null hypothesis is rejected, highlighting that the proposed system (A) consistently outshines the competing approaches.

### 4.3. Results for Audio-Based PSAS

The audio-based pain sentiment analysis system (PSAS) uses a deep learning-based feature representation approach, as described in the above sections (Figure 5). This method involves extracting various audio-based features, including statistical audio features, mel-frequency cepstral coefficients (MFCCs), and spectral features, all obtained from each speech audio using the Librosa Python package [73]. The Librosa package is widely used for the analysis of audio, speech, and music. Specifically, 11 statistical features, 128 MFCC features, and 224 spectral features were derived from the audio data. During the experiments, each individual feature set, as well as combinations of these features, was tested by the use of machine learning classifiers such as Decision Tree, Logistic Regression, K-Nearest Neighbors, and Support Vector Machine (SVM), with 50–50% and 75–25% training–testing splits. These feature sets, both individually and in combination, were also applied within the deep learning-based feature representation framework. Table 11 summarizes the performance results of the proposed audio-based pain sentiment recognition system when evaluated with these machine learning classifiers.

**Figure 12:**
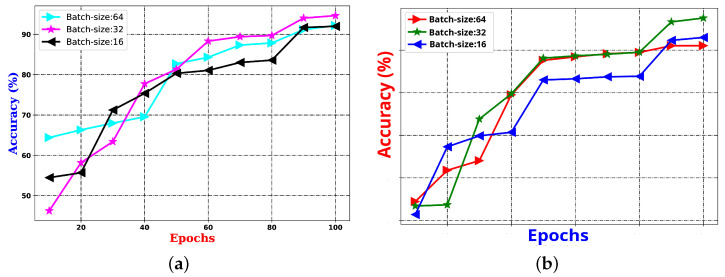
The performance outcome of the proposed pain SAS using audio features with (**a**) 50–50% training–testing, and (**b**) 75–25% training–testing sets.

The performance results displayed in Table 11 indicate that, in most cases, the Decision Tree classifier achieved the highest accuracy across various feature types for both the 50–50% and 75–25% training–testing splits. Moreover, it was found that MFCC and spectral features had a greater impact on performance than the statistical audio frequency features. Consequently, the 128 MFCC and 224 spectral features were combined to form a 352-dimensional feature vector for every speech audio sample. Then, this vector was utilized as input for the proposed deep learning architecture in the sentiment analysis system based on audio data. The deep neural network experiment was carried out with a batch size of 32, and the number of epochs ranged from 1 to 50. The performance outcome of the proposed PSAS is presented in Figure 12, where the results show that the optimal performance was achieved with a batch size of 32 and 100 epochs. Based on these results, these settings for batch size and epochs were selected for further experimentation with the audio-based PSAS.

**Figure 13:**
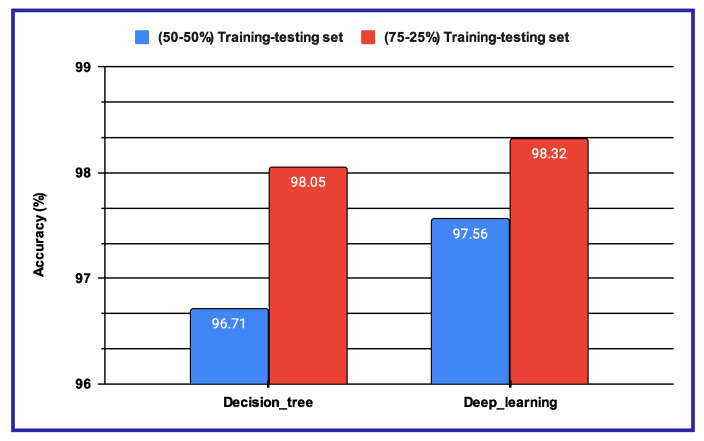
Performance of the proposed pain sentiment analysis system using the performance reported in Table 11 and Figure 12.

The performance results shown in Table 11 and Figure 12 reveal that, when using machine learning classifiers, the Decision Tree outperforms all other classifiers across the various audio-based features. However, when deep features are utilized, as illustrated in Figure 12a,b, the proposed sentiment analysis system based on audio data demonstrates enhanced performance when using a batch size of 32 and the number of epochs is 100. The results of these two experimental setups are further compared in Figure 13, where the performance of both the Decision Tree and deep learning-based methods is evaluated under different training–testing protocols. As seen in Figure 13, the features extracted using deep learning models provide better performance outcomes for the proposed audio-based PSAS.

### 4.4. Results for Multimodal PSAS

**Figure 14:**
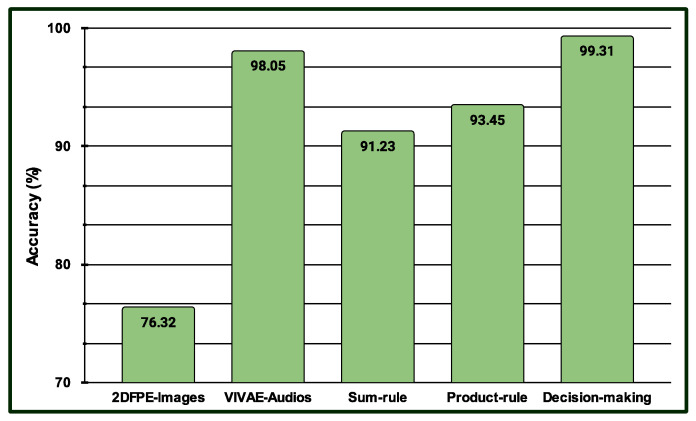
Performance of the proposed multimodal pain SAS ($MSAS_{1}$) using 2-class 2DFPE and VIVAE databases.

**Figure 15:**
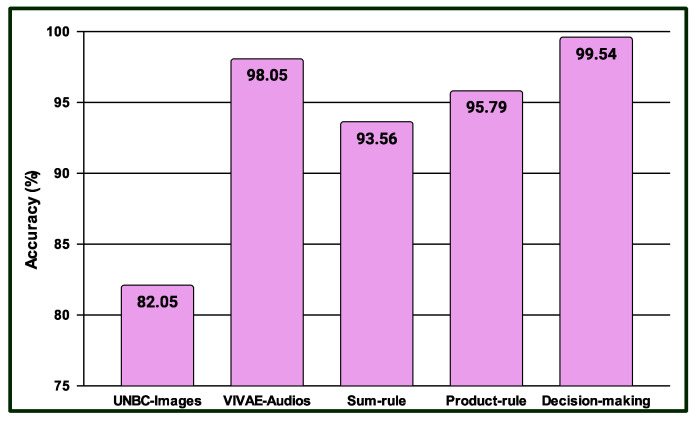
Performance of the proposed multimodal pain SAS ($MSAS_{2}$) using 3-Class UNBC-McMaster and VIVAE databases.

**Figure 16:**
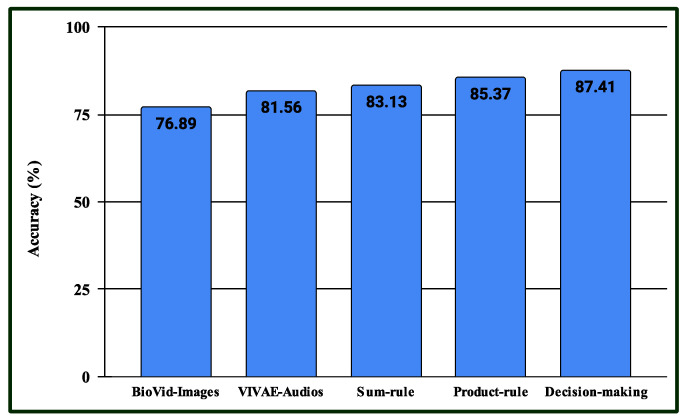
Performance of the proposed multimodal pain SAS ($MSAS_{3}$) using 4-class BioVid and VIVAE databases.

Three experiments are conducted in the multimodal PSAS. In the case of the first experiment, the classification scores of 149 NAG-class image samples from the 2DFPE database are fused with 149 NAG-class audio samples from the VIVAE database. Similarly, the classification scores of 136 OAG-class image samples from 2DFPE are fused with 136 OAG-class audio samples from VIVAE to asses the performance outcome of the proposed multimodal PSAS using the 2DFPE and VIVAE databases. In the second experiment, the classification scores of 265 NAG-class image specimens from the UNBC database are fused with 265 NAG-class audio samples from VIVAE; the classification scores of 136 CAG-class image samples from UNBC-McMaster are fused with 136 CAG-class audio samples from VIVAE; and the classification scores of 141 OAG-class image samples from UNBC-McMaster are fused with 141 OAG-class audio samples from VIVAE to asses the performance outcome of the three-class multimodal PSAS using the UNBC-McMaster and VIVAE databases. In the third experiment, the classification scores of 260 pain-level-1-class image samples from the BioVid database are fused with 260 low-intensity-class audio samples from VIVAE; the classification scores of 260 pain-level-2-class image samples from BioVid are fused with 260 moderate-intensity-class audio samples from VIVAE; the classification scores of 260 pain-level-3-class image samples from BioVid are fused with 260 strong-intensity-class audio samples from VIVAE; and the classification scores of 260 pain-level-4-class image samples from BioVid are fused with 260 peak-intensity-class audio samples from VIVAE. For all experiments, a 50–50% training–testing protocol is employed. The performance of these multimodal sentiment analysis systems is shown in Figure 14, Figure 15, and Figure 16, respectively. These figures clearly show that the proposed system performs exceptionally well when using the decision-level (majority voting) fusion technique.

A computational complexity analysis of the proposed system is presented in Table 12. The analysis focuses on the different derived models for the image-based and audio-based pain sentiment analysis systems, including the number of parameters required for training these models and the time needed to detect pain based on the image and audio data of the patient. Pain sentiment analysis plays a crucial role in the context of electronic healthcare, which increasingly relies on e-mobile and smart healthcare systems. This research involves analyzing patients’ facial images and speech audio data to determine their pain levels. However, these data can be affected by various challenges. To improve performance, (i) the combined effect of image and audio data is utilized in the multimodal pain sentiment analysis system; and (ii) advanced technologies, such as deep learning algorithms and their variants, are employed in this work.

## 5. Conclusions

This paper introduces a multimodal sentiment analysis system designed for pain detection, which integrates facial expressions and speech-audio data within an IoT-enabled healthcare framework. The approach is organized into five key components: (i) preprocessing of the images for extracting the facial patterns as regions of interest; (ii) learning and classifying features from images using two separate CNN and ensembles of these CNN architectures; (iii) enhancement of CNN performance through transfer learning, fine-tuning, and progressive image resizing techniques; (iv) speech-audio feature learning and classification utilizing both handcrafted and deep features; and (v) the fusion of classification scores from both image-based and audio-based samples to improve the overall system performance. The experiments cover two classes (‘pain’ and ‘no pain’), using the 2DFPE and UNBC shoulder pain datasets; three classes (‘non-aggressive (NAG) (no pain)’, ‘covertly aggressive (CAG) (pain of low amount)’, using the three-class UNBC dataset based on image data and the audio-based datasets, and ‘overtly aggressive (OAG) (pain of high amount)’), and five-class (‘no pain’, ‘pain amount 1’, ‘pain amount 2’, ‘pain amount 3’, and ‘pain amount 4’) pain classification tasks using the BioVid heat pain dataset. The experimental results demonstrate that the proposed pain sentiment analysis system surpasses benchmark methods. Additionally, the fusion of image-based and audio-based samples significantly boosts performance for all categories of pain recognition classes under this study. This work also addresses challenges such as synchronizing data across images, videos of facial expressions, and audio by enabling simultaneous data acquisition from multiple modalities, which adds complexity to the system’s implementation. While deploying image- and audio-based technologies over the web is feasible, implementing them on hardware presents a considerable challenge. The incorporation of the proposed system into a smart healthcare framework can significantly improve m-health, e-health, and telemedicine services, particularly in quarantine times, like during the COVID-19 pandemic or other infectious illnesses, for remote monitoring and evaluation of patients’ pain levels, which lowers the risk of disease transmission and will enable the provision of prompt therapies without the need for physical contact.

## Figures and Tables

**Table 1:** List of abbreviations employed in this paper.

Term	Abbreviation
2D Face Set Database with Pain Expression	2DFPE
Convolutional neural network	CNN
Covertly aggressive	CAG
Fast Fourier transformation	FFT
Internet Of Things	IoT
Mel-frequency cepstral coefficients	MFCCs
Multimodal pain sentiment analysis system	MPSAS
Non-aggressive	NAG
Overtly aggressive	OAG
Pain sentiment analysis system	PSAS
Tree-structured part model	TSPM
University Of Northern British Columbia-McMaster Shoulder Pain Expression Archive Database	UNBC-McMaster
Variably Intense Vocalizations of Affect And Emotion	VIVAE

**Table 2:** Detailed description of the $CNN_{A}$ architecture, including layer-wise parameters, where $t_{1}=\frac{1}{t}$, $t_{2}=\frac{1}{t_{1}}$, $t_{3}=\frac{1}{t_{2}}$, $t_{4}=\frac{1}{t_{3}}$, $t_{5}=\frac{1}{t_{4}}$, $t_{6}=\frac{1}{t_{5}}$, and $t_{7}=\frac{1}{t_{6}}$, *P* be the number of pain-classes.

Layers	Outputshape	Parameters	Layers	Outputshape	Parameters
Block1 (Input image: (128, 128, 3))			Block5		
Convolution (3 × 3)	(t, t, 30)	840	Convolution (3 × 3)	(t4, t4, 120)	129,720
Maxpool (2 × 2)	(t1, t1, 30)	0	Maxpool (2 × 2)	(t5, t5, 120)	0
BatchNormalization	(t1, t1, 30)	120	BatchNormalization	(t5, t5, 120)	480
Activation (ReLU)	(t1, t1, 30)	0	Activation (ReLU)	(t5, t5, 120)	0
Dropout	(t1, t1, 30)	0	Dropout	(t5, t5, 120)	0
Block2			Block6		
Convolution (3 × 3)	(t1, t1, 60)	16,260	Convolution (3 × 3)	(t5, t5, 240)	259,440
Maxpool (2 × 2)	(t2, t2, 60)	0	Maxpool (2 × 2)	(t6, t6, 240)	0
BatchNormalization	(t2, t2, 60)	240	BatchNormalization	(t6, t6, 240)	960
Activation (ReLU)	(t2, t2, 60)	0	Activation (ReLU)	(t6, t6, 240)	0
Dropout	(t2, t2, 60)	0	Dropout	(t6, t6, 240)	0
Block3			Block7		
Convolution (3 × 3)	(t2, t2, 90)	48,690	Convolution (3 × 3)	(t6, t6, 512)	110,6432
Maxpool (2 × 2)	(t3, t3, 90)	0	Maxpool (2 × 2)	(t7, t7, 512)	0
BatchNormalization	(t3, t3, 90)	360	BatchNormalization	(t7, t7, 512)	2048
Activation (ReLU)	(t3, t3, 90)	0	Activation (ReLU)	(t7, t7, 512)	0
Dropout	(t3, t3, 90)	0	Dropout	(t7, t7, 512)	0
Block4			Flatten		
Convolution (3 × 3)	(t3, t3, 120)	97,320	Dense	1 × (t7 × t7 × 512)	262,656
Maxpool (2 × 2)	(t4, t4, 120)	0	BatchNormalization	1 × (t7 × t7 × 512)	2048
BatchNormalization	(t4, t4, 120)	480	Activation (ReLU)	1 × (t7 × t7 × 512)	0
Activation (ReLU)	(t4, t4, 120)	0	Dropout	1 × (t7 × t7 × 512)	0
Dropout	(t4, t4, 120)	0	Dense	1 × P (=5)	2565
Total Parameters for The Input Image Size					1,930,659
Total Number of Trainable Parameters:					1,927,291
Non-trainable params:					3368

**Table 3:** Detailed description of the $CNN_{B}$ architecture, including layer-wise parameters, where $t_{1}=\frac{1}{t}$, $t_{2}=\frac{1}{t_{1}}$, $t_{3}=\frac{1}{t_{2}}$, $t_{4}=\frac{1}{t_{3}}$, $t_{5}=\frac{1}{t_{4}}$, $t_{6}=\frac{1}{t_{5}}$, and $t_{7}=\frac{1}{t_{6}}$, *P* be the number of pain-classes.

Layers	Outputshape	Parameters	Layers	Outputshape	Parameters
Block1 (Input image: (192, 192, 3))			Block5		
Convolution (3 × 3)	(t, t, 30)	840	Convolution (3 × 3)	(t4, t4, 240)	259,440
Maxpool (2 × 2)	(t1, t1, 30)	0	Maxpool (2 × 2)	(t5, t5, 240)	0
BatchNormalization	(t1, t1, 30)	120	BatchNormalization	(t5, t5, 240)	960
Activation (ReLU)	(t1, t1, 30)	0	Activation (ReLU)	(t5, t5, 240)	0
Dropout	(t1, t1, 30)	0	Dropout	(t5, t5, 240)	0
Block2			Block6		
Convolution (3 × 3)	(t1, t1, 60)	16,260	Convolution (3 × 3)	(t5, t5, 480)	1,037,280
Maxpool (2 × 2)	(t2, t2, 60)	0	Maxpool (2 × 2)	(t6, t6, 480)	0
BatchNormalization	(t2, t2, 60)	240	BatchNormalization	(t6, t6, 480)	1920
Activation (ReLU)	(t2, t2, 60)	0	Activation (ReLU)	(t6, t6, 480)	0
Dropout	(t2, t2, 60)	0	Dropout	(t6, t6, 480)	0
Block3			Block7		
Convolution (3 × 3)	(t2, t2, 90)	48,690	Convolution (3 × 3)	(t6, t6, 1024)	4,424,704
Maxpool (2 × 2)	(t3, t3, 90)	0	Maxpool (2 × 2)	(t7, t7, 1024)	0
BatchNormalization	(t3, t3, 90)	360	BatchNormalization	(t7, t7, 1024)	4096
Activation (ReLU)	(t3, t3, 90)	0	Activation (ReLU)	(t7, t7, 1024)	0
Dropout	(t3, t3, 90)	0	Dropout	(t7, t7, 1024)	0
Block4			Flatten		
Convolution (3 × 3)	(t3, t3, 120)	97,320	Dense	1 × (t7 × t7 × 1024)	1,049,600
Maxpool (2 × 2)	(t4, t4, 120)	0	BatchNormalization	1 × (t7 × t7 × 1024)	4096
BatchNormalization	(t4, t4, 120)	480	Activation (ReLU)	1 × (t7 × t7 × 1024)	0
Activation (ReLU)	(t4, t4, 120)	0	Dropout	1 × (t7 × t7 × 1024)	0
Dropout	(t4, t4, 120)	0	Dense	1 × P (=5)	5125
Total Parameters for The Input Image Size					6,951,531
Total Number of Trainable Parameters:					6,945,395
Non-trainable params:					6136

**Table 4:** List of parameters required for the audio-based deep learning technique.

Layer	Output Shape	Feature Size	Parameters
Flatten	(1, *d*)	(1,d)	0
Dense	(1, 512)	(1, 512)	(1 + d) × 512
BatNorm	(1, 512)	(1, 512)	2048
ActRelu	(1, 512)	(1, 512)	0
Dropout	(1, 512)	(1, 512)	0
Dense	(1, 256)	(1, 256)	(1 + 512) × 256 = 131,328
BatNorm	(1, 256)	(1, 256)	1024
ActRelu	(1, 256)	(1, 256)	0
Dropout	(1, 256)	(1, 256)	0
Dense	(1, 128)	(1,128)	(1 + 256) × 128 = 32,896
BatNorm	(1, 128)	(1,128)	512
ActRelu	(1, 128	(1, 128)	0
Dropout	(1, 128)	(1, 128)	0
Dense	(1, 3)	(1, 3)	(128 + 1) × 3 = 387
Total No. of Parameters			168,195 + ((1 + d) × 512)

**Table 5:** The description of employed databases concerning image samples for various pain intensity classes.

Class	Samples	Demography
2DFPE Database (2 classes of pain level)		
No Pain ($P_{1}$)	298	13 female and 10 male participants, without mention, belongs to Scotland, UK
Pain ($P_{2}$)	298	
UNBC Database (2 classes of pain level)		
No Pain ($Q_{1}$)	40,029	Same as for UNBC Database (3 classes of pain level)
Pain ($Q_{2}$)	8369	
UNBC Database (3 classes of pain level)		
No (Zero Intensity) Pain (NP) ($R_{1}$)	40,029	12 male and 13 female participants, with ages ranging from 18 to 65 years, from Ontario, Canada, regions
Low-Intensity Pain (LP) ($R_{2}$)	2909	
High-Intensity Pain (HP) ($R_{3}$)	5460	
BioVid Heat Pain dataset (5 classes of pain level)		
No Pain ($S_{1}$)	6900	30 male and 30 female participants, healthy adult individuals aged 18 and above, diverse age range, Western, European, and German regions
Pain Amount 1 ($S_{2}$)	6900	
Pain Amount 2 ($S_{3}$)	6900	
Pain Amount 3 ($S_{4}$)	6900	
Pain Amount 4 ($S_{5}$)	6900	

**Table 10:** A quantitative comparison (in Acc. (%)) of the proposed method with other competing methods for the image-based PSAS experiment is presented, maintaining the hypotheses $H_{0}:\mu_{A}\leq \mu_{\left\{B,C,D,E,F,G,H,I\right\}}$ and $H_{1}:\mu_{A}>\mu_{\left\{B,C,D,E,F,G,H,I\right\}}$, where μ denotes the mean performance, σ represents the standard deviation of performance, and N=2 indicates the number of tests.

3-Class UNBC-McMaster						
Method	Set-1	Set-2	μ	σ	t-statistic, *p*-value	Remarks
Proposed (A)	85.21	81.89	79.05	3.1123	H0: Null hypothesis; H1: Alternative hypothesis	
Anay et al. [70] (B)	83.12	80.45	84.51	0.18	0.1942, 0.8639>α	H0 is accepted, A<B
HoG (C)	69.74	64.56	65.21	0.25	10.31, 0.0092<α	H0 is rejected, A>C
Inception-v3 (D)	74.31	72.61	71.39	2.42	7.54, 0.0171<α	H0 is rejected, A>D
LBP (E)	71.36	62.72	67.04	37.32	3.5674, 0.0351<α	H0 is rejected, A>E
Lucey et al. [60] (F)	82.78	80.45	80.21	12.33	7.54, 0.0171<α	H0 is rejected, A>F
ResNet50 (G)	76.72	75.91	76.32	0.33	4.23, 0.0257<α	H0 is rejected, A>G
VGG16 (H)	73.33	76.23	74.78	4.21	3.97, 0.0288<α	H0 is rejected, A>H
Werner et al. [71] (I)	75.89	74.52	75.20	0.94	4.64, 0.0216<α	H0 is rejected, A>I
2-Class UNBC-McMaster						
Method	Set-1	Set-2	μ	σ	t-statistic, *p*-value	Remarks
Proposed (A)	87.43	85.81	79.05	3.1123	H0: Null hypothesis; H1: Alternative hypothesis	
Anay et al. [70] (B)	82.17	84.29	84.21	2.14	4.4314, 0.0371<α	H0 is rejected, A<B
HoG (C)	76.33	75.01	74.96	0.25	10.31, 0.0092<α	H0 is rejected, A>C
Inception-v3 (D)	77.45	75.19	76.32	2.55	7.40, 0.0088<α	H0 is rejected, A>D
LBP (E)	73.23	76.93	73.81	3.70	7.39, 0.0178<α	H0 is rejected, A>E
Lucey et al. [60] (F)	82.19	81.89	82.04	0.04	5.55, 0.0154<α	H0 is rejected, A>F
ResNet50 (G)	79.31	78.05	78.68	0.79	7.73, 0.0081<α	H0 is rejected, A>G
VGG16 (H)	77.45	71.38	74.41	18.42	3.88, 0.0301<α	H0 is rejected, A>H
Werner et al. [71] (I)	74.38	73.67	74.03	0.25	14.24, 0.0024<α	H0 is rejected, A>I
2-Class 2DFPE						
Method	Set-1	Set-2	μ	σ	t-statistic, *p*-value	Remarks
Proposed (A)	77.41	81.91	79.66	10.12	H0: Null hypothesis; H1: Alternative hypothesis	
Anay et al. [70] (B)	75.67	76.06	75.87	0.08	1.68, 0.1174>α	H0 is accepted, A<B
HoG (C)	68.61	72.71	70.66	8.40	2.95, 0.0489<α	H0 is rejected, A>C
Inception-v3 (D)	63.89	65.07	64.48	0.70	6.52, 0.0113<α	H0 is rejected, A>D
LBP (E)	67.34	69.47	68.40	2.27	4.52, 0.0227<α	H0 is rejected, A>E
Lucey et al. [60] (F)	72.58	75.01	73.80	2.95	2.29, 0.0743>α	H0 is accepted, A>F
ResNet50 (G)	63.78	65.39	64.59	1.30	6.30, 0.0121<α	H0 is rejected, A>G
VGG16 (H)	61.98	64.14	63.06	2.33	6.65, 0.0109<α	H0 is rejected, A>H
Werner et al. [71] (I)	69.05	71.37	70.21	2.69	3.73, 0.0324<α	H0 is rejected, A>I
5-Class BioVid						
Method	Set-1	Set-2	μ	σ	t-statistic, *p*-value	Remarks
Proposed (A)	37.67	37.81	37.42	0.62	H0: Null hypothesis; H1: Alternative hypothesis	
Anay et al. [70] (B)	28.15	26.41	26.96	2.13	15.4437, 0.00005<α	H0 is rejected, A>B
HoG (C)	23.05	25.24	24.34	2.62	17.7968, 0.00002<α	H0 is rejected, A>C
Inception-v3 (D)	22.78	24.89	23.84	2.23	13.15, 0.0028<α	H0 is rejected, A>D
LBP (E)	25.89	26.31	26.10	0.09	52.58, 0.0001<α	H0 is rejected, A>E
Lucey et al. [60] (F)	29.78	29.15	29.46	0.20	25.64, 0.0007<α	H0 is rejected, A>F
ResNet50 (G)	28.56	29.28	28.92	0.26	24.049, 0.0008<α	H0 is rejected, A>G
VGG16 (H)	25.09	26.18	25.64	0.59	22.03, 0.0010<α	H0 is rejected, A>H
Werner et al. [71] (I)	31.34	32.17	31.76	0.34	14.22, 0.0024<α	H0 is rejected, A>I

**Table 11:** The performance outcome of the proposed pain sentiment analysis system based on audio data using various machine learning classifiers.

	50–50% Training–Testing Set				75–25% Training–Testing Set			
Statistical Features								
Classifier	Accuracy	Precision	Recall	F1-Score	Accuracy	Precision	Recall	F1-Score
Logistic Regression	49.56	24.76	49.56	33.52	50.21	25.12	50.21	33.43
K-Nearest Neighbors	75.11	58.17	75.11	64.47	52.41	50.43	50.63	49.22
Decision Tree	75.11	58.17	75.11	64.47	50.21	25.12	50.21	33.43
Support Vector Machine	49.56	24.76	49.56	33.52	50.21	25.14	50.21	33.43
MFCC features								
Logistic Regression	52.23	35.63	52.23	39.52	75.34	58.18	75.07	64.74
K-Nearest Neighbors	52.23	35.63	52.23	39.52	75.66	63.29	75.22	67.39
Decision Tree	95.18	93.52	92.38	92.73	97.57	96.45	95.81	95.08
Support Vector Machine	26.27	9.63	26.41	13.48	97.43	96.21	95.72	94.69
Spectra features								
Logistic Regression	49.71	33.12	49.76	39.44	75.18	58.37	75.18	64.77
K-Nearest Neighbors	75.13	58.17	75.21	64.53	75.18	53.37	75.18	64.77
Decision Tree	96.45	95.41	95.43	94.21	98.51	97.49	97.43	97.19
Support Vector Machine	96.31	95.23	94.24	93.19	75.18	58.37	75.18	64.77

**Table 12:** Computational complexity in *sec.* of the proposed pain detection systems.

Data Type	Image Classification		Audio Classification
CNN Models	$CNN_{A}$	$CNN_{B}$	$CNN_{Audio}$
Trainable Parameters	1,947,431	6,654,119	281,603
Non-Trainable Parameters	2944	4736	1792
Total Parameters	1,944,487	6,649,383	283,395
Time	1.04	1.06	0.43

## Data Availability

In this manuscript, the datasets used have been acquired under proper license agreements from the corresponding institutions, following the appropriate channels.

## References

[1] Ande R., Adebisi B., Hammoudeh M., Saleem J. (2020). Internet of Things: Evolution and technologies from a security perspective. Sustain. Cities Soc..

[2] Khang A. (2024). AI and IoT Technology and Applications for Smart Healthcare Systems.

[3] Aminizadeh S., Heidari A., Dehghan M., Toumaj S., Rezaei M., Navimipour N.J., Stroppa F., Unal M. (2024). Opportunities and challenges of artificial intelligence and distributed systems to improve the quality of healthcare service. Artif. Intell. Med..

[4] Muhammad G., Alsulaiman M., Amin S.U., Ghoneim A., Alhamid M.F. (2017). A facial-expression monitoring system for improved healthcare in smart cities. IEEE Access.

[5] Williams A.d. (2002). Facial expression of pain: An evolutionary account. Behav. Brain Sci..

[6] Payen J.F., Bru O., Bosson J.L., Lagrasta A., Novel E., Deschaux I., Lavagne P., Jacquot C. (2001). Assessing pain in critically ill sedated patients by using a behavioral pain scale. Crit. Care Med..

[7] McGuire B., Daly P., Smyth F. (2010). Chronic pain in people with an intellectual disability: Under-recognised and under-treated?. J. Intellect. Disabil. Res..

[8] Puntillo K.A., Morris A.B., Thompson C.L., Stanik-Hutt J., White C.A., Wild L.R. (2004). Pain behaviors observed during six common procedures: Results from Thunder Project II. Crit. Care Med..

[9] Herr K., Coyne P.J., Key T., Manworren R., McCaffery M., Merkel S., Pelosi-Kelly J., Wild L. (2006). Pain assessment in the nonverbal patient: Position statement with clinical practice recommendations. Pain Manag. Nurs..

[10] Twycross A., Voepel-Lewis T., Vincent C., Franck L.S., von Baeyer C.L. (2015). A debate on the proposition that self-report is the gold standard in assessment of pediatric pain intensity. Clin. J. Pain.

[11] Knox D., Beveridge S., Mitchell L.A., MacDonald R.A. (2011). Acoustic analysis and mood classification of pain-relieving music. J. Acoust. Soc. Am..

[12] Giordano V., Luister A., Reuter C., Czedik-Eysenberg I., Singer D., Steyrl D., Vettorazzi E., Deindl P. (2022). Audio Feature Analysis for Acoustic Pain Detection in Term Newborns. Neonatology.

[13] Oshrat Y., Bloch A., Lerner A., Cohen A., Avigal M., Zeilig G. Speech prosody as a biosignal for physical pain detection. Proceedings of the Conference on 8th Speech Prosody.

[14] Ren Z., Cummins N., Han J., Schnieder S., Krajewski J., Schuller B. Evaluation of the pain level from speech: Introducing a novel pain database and benchmarks. Proceedings of the Speech Communication; 13th ITG-Symposium.

[15] Ashraf A.B., Lucey S., Cohn J.F., Chen T., Ambadar Z., Prkachin K.M., Solomon P.E. (2009). The painful face–pain expression recognition using active appearance models. Image Vis. Comput..

[16] Lucey P., Cohn J., Howlett J., Lucey S., Sridharan S. (2011). Recognizing emotion with head pose variation: Identifying pain segments in video. IEEE Trans. Syst. Man Cybern.-Part B.

[17] Littlewort-Ford G., Bartlett M.S., Movellan J.R. Are your eyes smiling? Detecting genuine smiles with support vector machines and Gabor wavelets. Proceedings of the 8th Joint Symposium on Neural Computation.

[18] Umer S., Dhara B.C., Chanda B. (2019). Face recognition using fusion of feature learning techniques. Measurement.

[19] Bisogni C., Castiglione A., Hossain S., Narducci F., Umer S. (2022). Impact of deep learning approaches on facial expression recognition in healthcare industries. IEEE Trans. Ind. Inform..

[20] Cunningham P., Delany S.J. (2021). k-Nearest neighbour classifiers-A Tutorial. ACM Comput. Surv. (CSUR).

[21] Mohankumar N., Narani S.R., Asha S., Arivazhagan S., Rajanarayanan S., Padmanaban K., Murugan S. (2025). Advancing chronic pain relief cloud-based remote management with machine learning in healthcare. Indones. J. Electr. Eng. Comput. Sci..

[22] Anderson K., Stein S., Suen H., Purcell M., Belci M., McCaughey E., McLean R., Khine A., Vuckovic A. (2025). Generalisation of EEG-Based Pain Biomarker Classification for Predicting Central Neuropathic Pain in Subacute Spinal Cord Injury. Biomedicines.

[23] Hu F., Xia G.S., Hu J., Zhang L. (2015). Transferring deep convolutional neural networks for the scene classification of high-resolution remote sensing imagery. Remote Sens..

[24] Yadav A., Vishwakarma D.K. (2020). A comparative study on bio-inspired algorithms for sentiment analysis. Clust. Comput..

[25] Nugroho H., Harmanto D., Al-Absi H.R.H. On the development of smart home care: Application of deep learning for pain detection. Proceedings of the 2018 IEEE-EMBS Conference on Biomedical Engineering and Sciences (IECBES).

[26] Haque M.A., Bautista R.B., Noroozi F., Kulkarni K., Laursen C.B., Irani R., Bellantonio M., Escalera S., Anbarjafari G., Nasrollahi K. Deep multimodal pain recognition: A database and comparison of spatio-temporal visual modalities. Proceedings of the 2018 13th IEEE International Conference on Automatic Face & Gesture Recognition (FG 2018).

[27] Menchetti G., Chen Z., Wilkie D.J., Ansari R., Yardimci Y., Çetin A.E. Pain detection from facial videos using two-stage deep learning. Proceedings of the 2019 IEEE Global Conference on Signal and Information Processing (GlobalSIP).

[28] Yu I.C., Guo J.M., Lin W.C., Fang J.T. (2025). Development of nonverbal communication behavior model for nursing students based on deep learning facial expression recognition technology. Cogent Educ..

[29] Kasundra A., Chanchlani R., Lal B., Thanveeru S.K., Ratre G., Ahmad R., Sharma P.K., Agrawal A., Kasundra Sr A. (2025). Role of Artificial Intelligence in the Assessment of Postoperative Pain in the Pediatric Population: A Systematic Review. Cureus.

[30] M. Al-Eidan R., Al-Khalifa H., Al-Salman A. (2020). Deep-learning-based models for pain recognition: A systematic review. Appl. Sci..

[31] Gouverneur P., Li F., Adamczyk W.M., Szikszay T.M., Luedtke K., Grzegorzek M. (2021). Comparison of feature extraction methods for physiological signals for heat-based pain recognition. Sensors.

[32] Pikulkaew K., Boonchieng E., Boonchieng W., Chouvatut V. (2021). Pain detection using deep learning with evaluation system. Proceedings of the Fifth International Congress on Information and Communication Technology.

[33] Ismail L., Waseem M.D. (2023). Towards a Deep Learning Pain-Level Detection Deployment at UAE for Patient-Centric-Pain Management and Diagnosis Support: Framework and Performance Evaluation. Procedia Comput. Sci..

[34] Wu J., Shi Y., Yan S., Yan H.M. (2024). Global-Local Combined Features to Detect Pain Intensity from Facial Expression Images with Attention Mechanism. J. Electron. Sci. Technol..

[35] Othman E., Werner P., Saxen F., Al-Hamadi A., Gruss S., Walter S. (2023). Classification networks for continuous automatic pain intensity monitoring in video using facial expression on the X-ITE Pain Database. J. Vis. Commun. Image Represent..

[36] Thiam P., Kessler V., Walter S., Palm G., Schwenker F. (2017). Audio-visual recognition of pain intensity. Proceedings of the IAPR Workshop on Multimodal Pattern Recognition of Social Signals in Human-Computer Interaction.

[37] Hossain M.S. (2016). Patient state recognition system for healthcare using speech and facial expressions. J. Med Syst..

[38] Zeng Z., Pantic M., Roisman G.I., Huang T.S. A survey of affect recognition methods: Audio, visual and spontaneous expressions. Proceedings of the 9th International Conference on Multimodal Interfaces.

[39] Thiam P., Bellmann P., Kestler H.A., Schwenker F. (2019). Exploring deep physiological models for nociceptive pain recognition. Sensors.

[40] Krizhevsky A., Sutskever I., Hinton G.E. (2012). Imagenet classification with deep convolutional neural networks. Adv. Neural Inf. Process. Syst..

[41] Dashtipour K., Gogate M., Cambria E., Hussain A. (2021). A novel context-aware multimodal framework for persian sentiment analysis. arXiv.

[42] Sagum R.A. (2021). An Application of Emotion Detection in Sentiment Analysis on Movie Reviews. Turk. J. Comput. Math. Educ. (TURCOMAT).

[43] Rustam F., Khalid M., Aslam W., Rupapara V., Mehmood A., Choi G.S. (2021). A performance comparison of supervised machine learning models for Covid-19 tweets sentiment analysis. PLoS ONE.

[44] Liu Z.x., Zhang D.g., Luo G.z., Lian M., Liu B. (2020). A new method of emotional analysis based on CNN–BiLSTM hybrid neural network. Clust. Comput..

[45] Ridouan A., Bohi A., Mourchid Y. (2025). Improving Pain Classification using Spatio-Temporal Deep Learning Approaches with Facial Expressions. arXiv.

[46] Zhu X., Ramanan D. Face detection, pose estimation, and landmark localization in the wild. Proceedings of the 2012 IEEE Conference on Computer Vision and Pattern Recognition.

[47] Hossain S., Umer S., Rout R.K., Tanveer M. (2023). Fine-grained image analysis for facial expression recognition using deep convolutional neural networks with bilinear pooling. Appl. Soft Comput..

[48] Szegedy C., Liu W., Jia Y., Sermanet P., Reed S., Anguelov D., Erhan D., Vanhoucke V., Rabinovich A. Going deeper with convolutions. Proceedings of the IEEE Conference on Computer Vision and Pattern Recognition.

[49] Umer S., Rout R.K., Pero C., Nappi M. (2021). Facial expression recognition with trade-offs between data augmentation and deep learning features. J. Ambient. Intell. Humaniz. Comput..

[50] Saxena A. (2016). Convolutional neural networks: An illustration in TensorFlow. XRDS: Crossroads, ACM Mag. Stud..

[51] Lawrence S., Giles C.L., Tsoi A.C., Back A.D. (1997). Face recognition: A convolutional neural-network approach. IEEE Trans. Neural Netw..

[52] Martin E., d’Autume M.d.M., Varray C. (2012). Audio Denoising Algorithm with Block Thresholding. Image Process..

[53] Fu Z., Lu G., Ting K.M., Zhang D. (2010). A survey of audio-based music classification and annotation. IEEE Trans. Multimed..

[54] Logan B. (2000). Mel Frequency Cepstral Coefficients for Music Modeling. ISMIR.

[55] Lee C.H., Shih J.L., Yu K.M., Lin H.S. (2009). Automatic music genre classification based on modulation spectral analysis of spectral and cepstral features. IEEE Trans. Multimed..

[56] Nawab S., Quatieri T., Lim J. (1983). Signal reconstruction from short-time Fourier transform magnitude. IEEE Trans. Acoust. Speech Signal Process..

[57] Gulli A., Pal S. (2017). Deep Learning with Keras.

[58] Bergstra J., Breuleux O., Bastien F., Lamblin P., Pascanu R., Desjardins G., Turian J., Warde-Farley D., Bengio Y. Theano: A CPU and GPU math expression compiler. Proceedings of the Python for Scientific Computing Conference (SciPy).

[59] Holz N., Larrouy-Maestri P., Poeppel D. (2022). The variably intense vocalizations of affect and emotion (VIVAE) corpus prompts new perspective on nonspeech perception. Emotion.

[60] Lucey P., Cohn J.F., Prkachin K.M., Solomon P.E., Matthews I. Painful data: The UNBC-McMaster shoulder pain expression archive database. Proceedings of the 2011 IEEE International Conference on Automatic Face & Gesture Recognition (FG).

[61] 2D Face Dataset with Pain Expression. http://pics.psych.stir.ac.uk/2D_face_sets.htm.

[62] Walter S., Gruss S., Ehleiter H., Tan J., Traue H.C., Werner P., Al-Hamadi A., Crawcour S., Andrade A.O., da Silva G.M. The biovid heat pain database data for the advancement and systematic validation of an automated pain recognition system. Proceedings of the 2013 IEEE International Conference on Cybernetics (CYBCO).

[63] Ekman P. (1992). An Argument for Basic Emotions. Cogn. Emot..

[64] Mollahosseini A., Hasani B., Mahoor M.H. (2017). Affectnet: A database for facial expression, valence, and arousal computing in the wild. IEEE Trans. Affect. Comput..

[65] Umer S., Dhara B.C., Chanda B. (2018). An iris recognition system based on analysis of textural edgeness descriptors. IETE Tech. Rev..

[66] Umer S., Dhara B.C., Chanda B. Biometric recognition system for challenging faces. Proceedings of the 2015 Fifth National Conference on Computer Vision, Pattern Recognition, Image Processing and Graphics (NCVPRIPG).

[67] Szegedy C., Vanhoucke V., Ioffe S., Shlens J., Wojna Z. Rethinking the inception architecture for computer vision. Proceedings of the IEEE Conference on Computer Vision and Pattern Recognition.

[68] McNeely-White D., Beveridge J.R., Draper B.A. (2020). Inception and ResNet features are (almost) equivalent. Cogn. Syst. Res..

[69] Simonyan K., Zisserman A. (2014). Very deep convolutional networks for large-scale image recognition. arXiv.

[70] Ghosh A., Umer S., Khan M.K., Rout R.K., Dhara B.C. (2023). Smart sentiment analysis system for pain detection using cutting edge techniques in a smart healthcare framework. Clust. Comput..

[71] Werner P., Al-Hamadi A., Limbrecht-Ecklundt K., Walter S., Gruss S., Traue H.C. (2016). Automatic pain assessment with facial activity descriptors. IEEE Trans. Affect. Comput..

[72] Gibbons J.D. (1993). Nonparametric Statistics: An Introduction.

[73] McFee B., Raffel C., Liang D., Ellis D.P., McVicar M., Battenberg E., Nieto O. librosa: Audio and music signal analysis in python. Proceedings of the 14th Python in Science Conference (SCIPY 2015).

